# Bidirectional relations between the maternal and infant gut microbiome and behavior

**DOI:** 10.1038/s41390-025-04630-9

**Published:** 2025-12-12

**Authors:** Caroline Kelsey, Robert Moulder, Heath Yancey, Stephanie Prescott, John A. McCulloch, Giorgio Trinchieri, Caitlin Dreisbach, Jeanne Alhusen, Tobias Grossmann

**Affiliations:** 1 Department of Psychology, University of Virginia, Charlottesville, VA, USA.; 2 Department of Pediatrics, Division of Developmental Medicine, Boston Children’s Hospital, Boston, MA, USA.; 3 Department of Psychology, University of Illinois (Urbana-Champaign), Champaign, IL, USA.; 4 Institute of Cognitive Science, University of Colorado Boulder, Boulder, CO, USA.; 5 National Cancer Institute, National Institutes of Health, Bethesda, MD, USA.; 6 Fairfax Neonatal Associates, Inova Children’s Hospital, Falls Church, VA, USA.; 7 School of Nursing, University of Virginia, Charlottesville, VA, USA.; 8 School of Nursing, University of Rochester, Rochester, NY, USA.; 9 Goergen Institute of Data Science, University of Rochester, Rochester, NY, USA.

## Abstract

**BACKGROUND:**

An infant’s mother is one of the first sources of neonatal microbial colonization, and infant-maternal dyad microbial variations have been linked to childhood behavioral traits and mental health outcomes. However, how the gut microbiome influences mental health, including potential bidirectional relations between mother and child, remains poorly understood.

**METHOD:**

Using metagenomic sequencing and behavioral questionnaires, we examined within-person and between-person (mother-infant dyad) associations between the gut microbiota and behavior across the first year of postnatal life (*N* = 121 dyads; *N* = 514 stool samples).

**RESULTS:**

There were rapid changes in taxa diversity and gut microbiota composition for infants, whereas the maternal microbiome remains relatively constant. Gut microbes and functional terms (e.g., antibiotic resistance genes and virulence factors) were associated with infant temperament but not maternal depression symptoms. Whereas maternal depression was not associated with any maternal taxa or functional terms.

**CONCLUSIONS:**

Our findings provide evidence for complex within- and between-person relations between maternal and infant gut microbiomes and behavioral traits.

Microbial colonization contributes to neuronal, hormonal, and immune signaling, forming what is known as the gut-brain axis, making the gut microbiota critical to neurodevelopment and adult mental health.^1,2^ One of the first sources of neonatal microbial colonization is an infant’s mother.^3^ Before delivery, the mother’s microbiome may influence fetal immune and metabolic programming.^4^ At birth, mothers share microorganisms with their newborn infants through processes such as delivery, breastfeeding, and close physical contact.^5–7^ Much less is known about the role that the mothers’ microbiome plays in infant colonization and behavioral development beyond birth.^8,9^ In addition, little is known about the microbial underpinnings of postpartum depression. Understanding microbial contributions to infant behavior and maternal mental health promises to shed light on key developmental and biological processes.^10^

In human adults, the relation between the number of microorganisms and depression is unclear, as both increased and decreased microbial diversity and individual microbes (e.g., *Bifidobacterium* and *Lactobacillus*) have been linked to major depressive disorder.^11–14^ It is also unknown as to whether individuals with postpartum and major depressive disorder show similar microbial patterns. Overall, the association between the gut microbiome and postpartum depression is critical to characterize, given the high prevalence of postpartum depression (10% of mothers meet criteria for postpartum depression) and the importance of maternal mental health to both maternal and child well-being.^15^ More specifically, depressed mothers may be less sensitive to their infant’s cues and may exhibit less positive emoting when interacting with their infants.^16^ These behaviors in turn can impact the infant’s temperament, with infants of depressed mothers being reported as fussier and more fearful.^16^ However, the findings on the link between maternal depressive symptoms and infant behavioral temperament are mixed.^17^ Therefore, this association may be clarified by considering other intermediary factors such as the gut microbiota.

For infants, associations between the microbiome and behavior emerge early in life, with several cross-sectional studies reporting associations between the microbiome and infant affective and regulatory development.^18–26^ For example, increased taxa diversity, or total relative abundance and evenness of microorganisms in the gut microbiome, has been linked to decreased negative emotionality, increased internalizing symptoms, and decreased cognitive abilities.^18–21,27^ Previous work has shown that there is no association between taxa diversity and temperament within the first month of postnatal life.^21^ Nonetheless, the study found specific infant gut microbial species, such as *Bifidobacterium pseudocatenulatum, Streptococcus vestibularis*, and *Thermovibrio guaymasensis*, and certain virulence factors were linked to individual differences in infant behavioral temperament.^21^

Studying the microbe-behavior associations across the first few months of life is especially important as this is a time of tremendous growth for both the microbiome and behavioral temperament.^28,29^ Bifidobacterium is the predominant microbe type during the first month of life.^30^ Then, with the introduction of solid foods, Bacteroidota and Firmicutes become increasingly present. For behavioral temperament, similar transitions happen where behavioral repertoires are limited during the first few months of life, and by the second half of the first year, behaviors such as fear, attentional control, and self-soothing emerge and become more prominent during this time.^31,32^

Critically, there is an overlap between how maternal depressive symptoms impact infant behavioral temperament and how maternal depressive symptoms impact the maternal and infant microbiota. For example, altered diet,^33^ breastfeeding initiation and duration,^34,35^ infection and antibiotic treatment,^36,37^ and outside social and environmental contact^9,38,39^ have been linked with changes in infant temperament and maternal mood. There may also be similar bidirectional processes by which infant behavioral temperament may influence maternal mood, maternal microbiota, and their own (infant) microbiota.^40^ For example, previous behavioral work shows bi-directional longitudinal associations between temperament and parental care.^41,42^ In addition, the amount of time the infant and mother spend together impacts the development of the infanťs gut microbiome.^43^ Both decreased maternal sensitivity and decreased infant negative emotionality are associated with infant antibiotic administration.^21,44^ Further, antibiotic administration is linked to decreases in taxa diversity, increases in antibiotic resistance genes in the gut, and elevated levels of depression in human and animal models.^45^ However, more work needs to be done to understand how the timing of antibiotic administration (e.g., during labor vs. postnatally) and who is receiving the antibiotics (infant vs mother) impacts the development of antibiotic resistance.

Using longitudinal data from mother-infant dyads, we characterized within-individual trajectories for total gut microbiome taxonomic diversity (chao1) and at the genera level at 1, 7, and 14 months after delivery. This study aimed to assess (1) the within-person (infant-infant and mother-mother) associations (i.e., consistency) between the gut microbiota and behavior across the first year of the infant’s life, and (2) the between-person (mother-infant) dyad associations between the gut microbiota and behavior across the first year of the infant’s life. For each aim, we took two approaches to characterizing the gut microbiome: (1) examining gut microbiome alpha diversity (chao1 and Shannon; note additional analyses with Faith’s phylogenetic diversity are in supplementary materials) and (2) identifying individual microbes that are associated with the behaviors of interest. We hypothesized that the microbiome composition would be linked to behavior within individuals and between mother-infant dyads (behavioral temperament for infants and maternal depression for mothers). In addition, we assessed the associations between maternal depressive symptoms and behavioral temperament. Here, we hypothesized that maternal depressive symptoms would be associated with greater negative emotionality and decreased regulation.

## METHOD

Mother and infant dyads (*N* = 121) participated in the present study and contributed a total of 514 stool samples. Families came to the lab when their infant was around 1 month, 7 months, and 14 months. Participants were recruited from a local hospital and are representative of the surrounding Mid-Atlantic college town (for socio-demographic information, see Table 1). To be included in the analysis, mothers or infants needed to contribute a stool sample or mental health information from at least one time point (see Supplementary Tables S13, 14 for information on sample contribution and sample size for each data type). Note, maternal tobacco use and substance use was not collected or screened for. All infants were born near term (>36 weeks) and none had low birthweight (i.e., all infants weighed >2500 grams at birth). Subject recruitment happened between April 2, 2018, through May 25, 2019. Parents gave informed written consent for themselves and their infants to participate following the Declaration of Helsinki, and families received payment for their participation. All procedures were approved by and carried out following the University of Virginia’s Institutional Review Board.

### Stool collection and processing

Parents were instructed to collect their stool and their infanťs stool samples at home using a sterile biospecimen container and infant diapers, respectively. See ref.^21^ for a previous study using this protocol and supplementary methods for more details on the stool processing.

### Shotgun metagenomic analysis

The shotgun sequencing output was analyzed using a series of pipelines and functions in the R language developed in-house and publicly available on GitHub under the package name Just Another Microbiology System (JAMS), found at.^46^ Details of this method can be found in supplementary materials and other manuscripts.^47–49^ The output of shotgun metagenomic sequencing included both taxonomic and functional characterization of the microbiota (functional terms assessed: Gene Ontology (GO) terms [describe the molecular function, cellular component, and biological process of genes], virulence factor genes [describe the potential pathogenicity of the microbes], and antibiotic resistance genes).

### Infant temperament

Infant behavioral temperament was assessed using parental reports from the 91-item Infant Behavior Questionnaire Revised Short Form IBQ-R;^50,51^ at the 1-month and 7-month time point, and the 107-item Early Childhood Behavior Questionnaire Short Form ECBQ-R;^52^ at the 14-month time point. Parents completed the questionnaire online using the Qualtrics platform before their appointment. Scores for negative emotionality and regulation were computed and included in the main analyses. For descriptive information on the subscales and results with urgency, see supplementary materials.

### Maternal depressive symptoms

Mothers completed the Edinburgh Postnatal Depression Scale, a 10-item self-report questionnaire measuring the level of depressive and anxiety symptoms at each time point.^53,54^ Mothers rated a series of symptoms and feelings on a four-point intensity scale (0–3). Item scores were totaled with higher scores reflecting greater levels of depressive symptoms (possible score range 0–30; T1: *M* = 5.29; *SD* = 4.30; T2: *M* = 4.71, *SD* = 5.25; T3: *M* = 5.33, *SD* = 4.91). Several mothers had scores with concerning depressive symptomology levels (a score of ≥13; T1: *n* = 7; T2 *n* = 6; T3 *n* = 6).

### Statistical analysis

Spearman’s rank correlations were conducted between sociodemographic information, health and delivery information, gut microbiome compositions, maternal depressive symptoms, and infant behavioral temperament (see Supplementary Figs. S1–6). Analyses were conducted using R.^55^

Principal component analysis (PCA; psych package in R) was used as a dimension reduction technique to allow for the maximal inclusion of covariates in subsequent analyses (see supplementary methods and Supplementary Table S1).

To assess group-level changes in Chao1 LKT over time for mothers and infants, a linear mixed effects model (lme4) package^56^ using restricted maximum likelihood estimates was conducted. This model included fixed effects of time (T1, T2, T3), individual (mother, infant), covariates (PC1, PC2, PC3, and PC4), and time-by-individual interaction, and a random intercept for each subject. Significant interaction effects were probed using pairwise comparisons with Bonferroni-adjusted *p*-values. Additional analyses examining other alpha taxa diversity metrics (Shannon and Faith’s PD) and functional term relative abundance (virulence factors, antibiotic resistance genes, and GO Terms are in supplementary results. Note, in line with the analysis approach of previous work (Kelsey et al., 2021) and to reflect the notion that it is the number of functional terms that has an impact on biology only Chao1 (richness) of the functional terms was considered.

Bayesian random intercept cross-lagged panel models (BRI-CLPMs) were conducted using the JAGS software for Bayesian analysis, and the rjags package in R.^57^ Cross-lagged (between-person) and autoregressive (within-person) effects were free parameters. The model was designed to examine longitudinal associations; therefore, within time point associations were not assessed. This decision also helped with reducing free parameters and model convergence (see Supplementary Figs. S1–6 showing within time point associations and Supplementary Fig. S7 showing between time point associations). All autoregressive and cross-lagged parameters were estimated with uninformative uniform priors, whereas all variance components were estimated with informative inverse gamma priors. Statistical significance was assessed via a 95% Bayesian credible interval for each parameter. First, BRI-CLPM models were conducted to assess the relation between mother and infant gut microbiome taxa diversity (Chao1 LKT) and mental health outcomes (depressive symptoms and behavioral temperament [negative emotionality and regulation separately]). This process was then repeated for virulence factors, antibiotic resistance genes, and GO Terms relative abundance (Chao1) metrics (see Supplementary Tables S2–9). All models controlled for PC1, PC2, PC3, and PC4.

Microbiome Multivariate Association with Linear Models (Maaslin2) was used to identify potential microbial biomarkers of infant temperament and maternal depression. Analyses were conducted separately for infants’ and mothers’ microbial taxa and features. For these models, infant negative affectivity, infant regulation, maternal depression, age in months at assessment, and principal components (PC1:PC4) were entered as fixed effects, and subject was entered as a random effect. For analysis with Maaslin2 the following options were used: minimum abundance = 0.01, minimum prevalence = 10%, normalization = TSS, transformation = Log, BH qvalue threshold = 0.05).

## RESULTS

### Infants, but not postpartum mothers, exhibit group-level changes in taxa diversity across the first year of the infant’s life

**Fig. 1:**
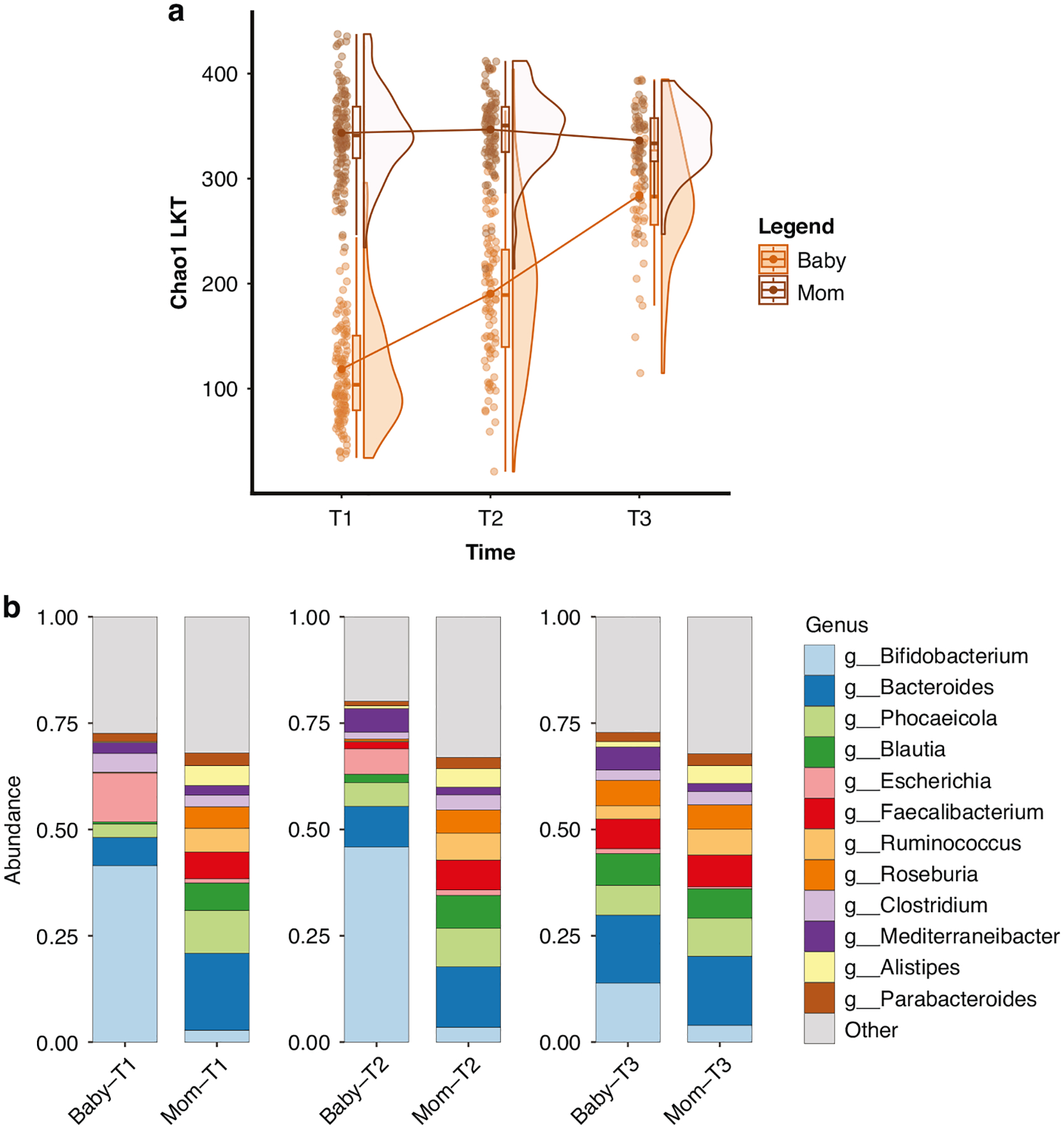
Mother and infant (*N* = 121) microbiota development across the first year of the infant’s life. Mother and infant (*N* = 121) taxa richness (Chao1 LKT; **a**) and top 12 genera relative abundances (**b**) T1 is approximately 1 month, T2 is approximately 7 months, and T3 is approximately 14 months. LKT Last known taxa, T Time.

There was a significant individual-by-time interaction effect (*B* = −83.26, *SE* = 5.66, *t* = −14.70, *p* < 0.001). Post hoc analyses revealed that infants’ diversity increased over time (all *p-values* < .001) but maternal Chao1 LKT levels were not significantly different over time (*p* = 1.00). Moreover, at all-time points, infants had significantly less diverse microbiomes than their mothers (all *p*-values < 0.001; see Fig. 1a; for functional terms see Supplementary Figs. S11–15). Genera relative abundance levels were computed for mothers and infants at each time point. Infants during the first seven months of life have a microbiome composition dominated by *Bifidobacterium* and *Escherichia*, and by 14 months they show increases in *Faecalibacterium* and *Roseburia* and have gut microbiome compositions similar to their mothers (Fig. 1b).

### Within-individual associations between taxa diversity and behavior

#### Mothers and infants have consistent taxa richness and behavioral outcomes throughout the first year of the infant’s life.

**Fig. 2:**
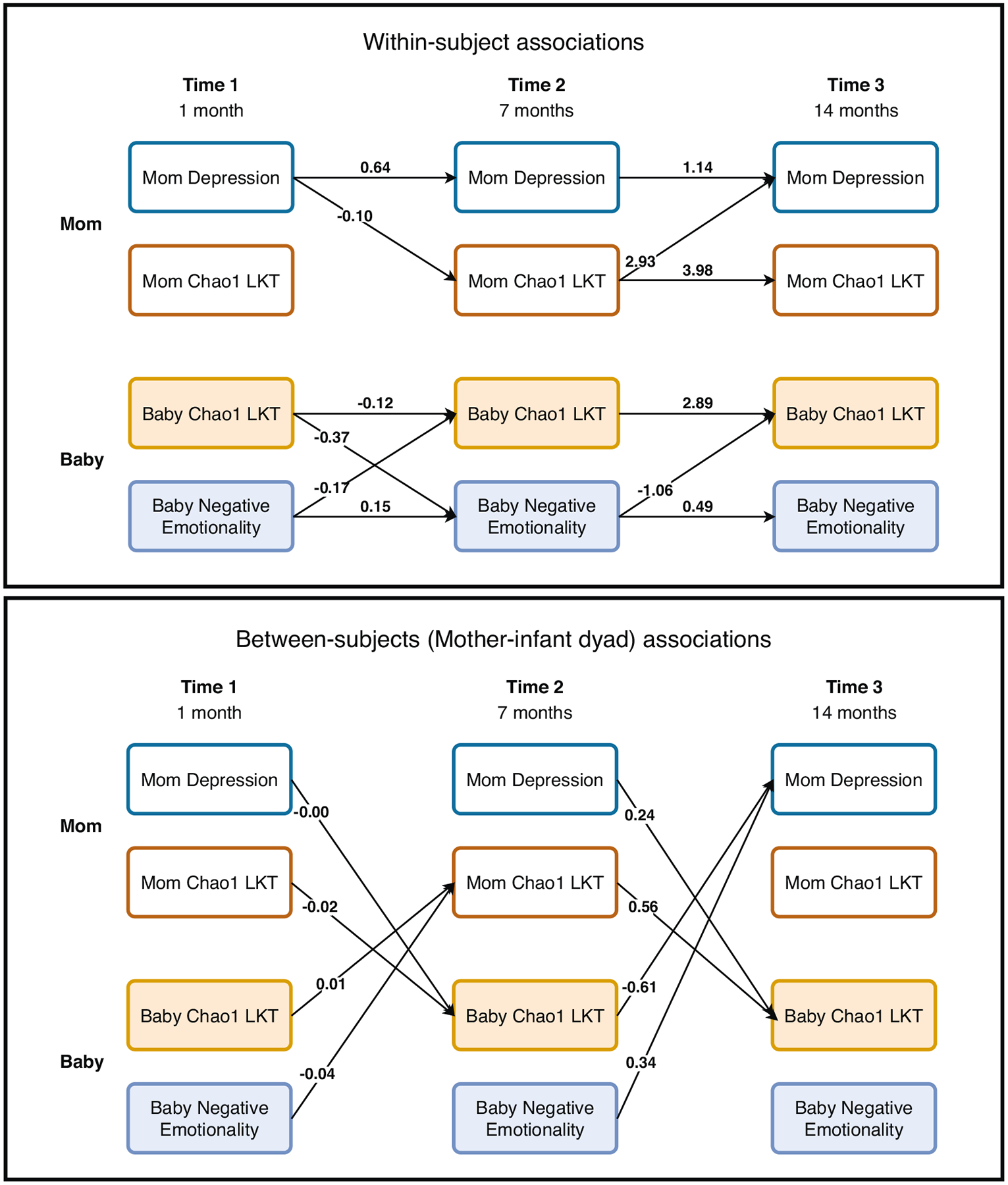
Path diagram for mother and infant (N = 121) taxa richness (chao1 LKT) and psychological outcomes (maternal depressive symptoms and negative emotionality). Results of the Bayesian random intercept cross-lagged panel models (BRI-CLPMs). Within-subject associations are infant to infant or mother to mother and between subject associations are between mother and infant. Only significant paths (CI does not contain zero) are shown above. See supplementary materials for all paths tested and all coefficient values. LKT Last known taxa.

**Fig. 3:**
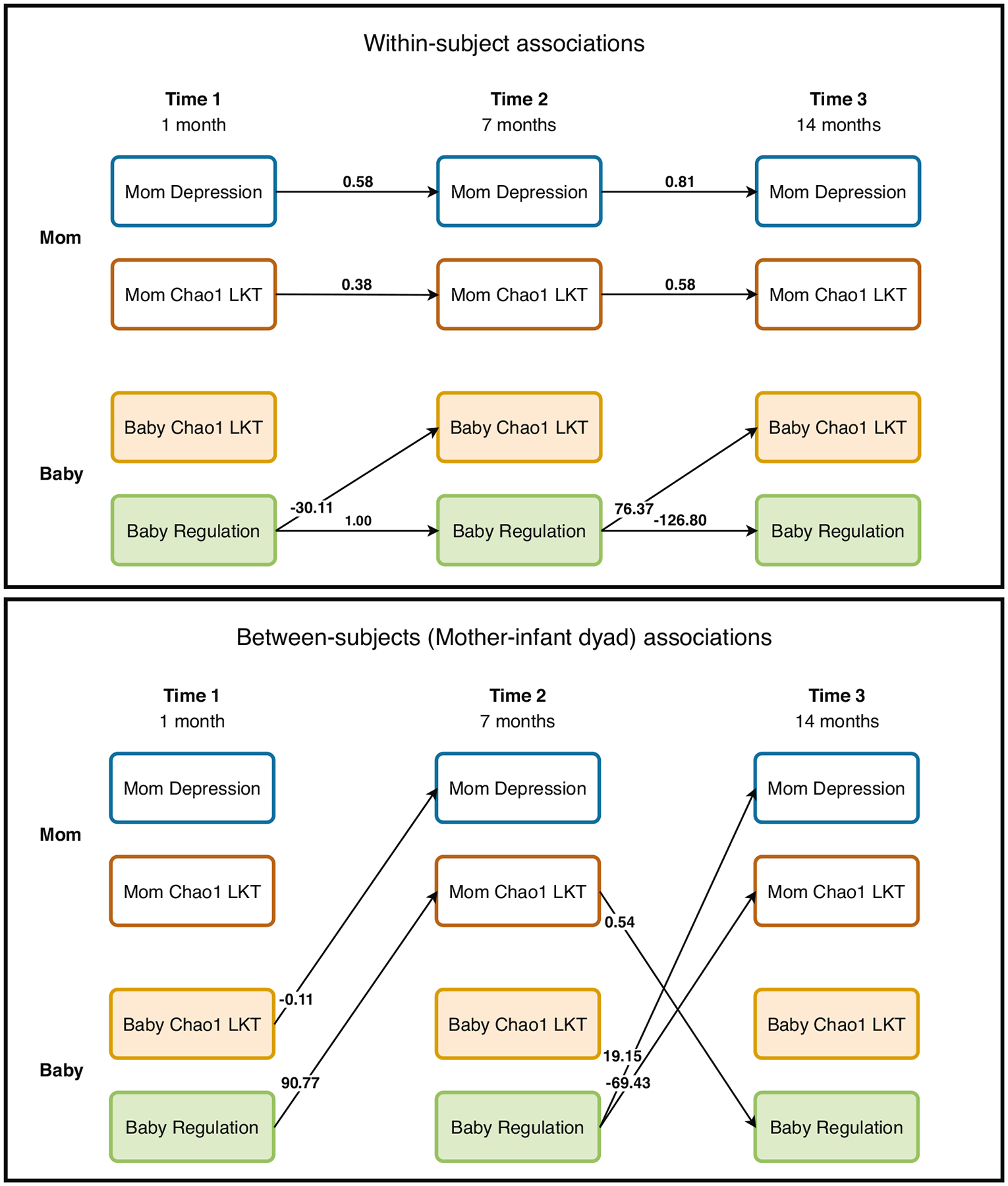
Path diagram for mother and infant (N = 121) taxa richness (chao1 LKT) and psychological outcomes (maternal depressive symptoms and regulation). Results of the Bayesian random intercept cross-lagged panel models (BRI-CLPMs). Within-subject associations are infant to infant or mother to mother and between subject associations are between mother and infant. Only significant paths (CI does not contain zero) are shown above. See supplementary materials for all paths tested and all coefficient values.

There was a significant degree of within-person consistency such that infant Chao1 LKT at earlier time points was significantly associated with infant Chao1 LKT at later time points (T1 to T2 B_neg_ = −0.12, T2 to T3 B_neg_ = 2.89; note this association was not found in the regulation model) and maternal Chao1 LKT at earlier time points was significantly associated with maternal Chao1 LKT at later time points (T1-T2 B_reg_ = .38, T2-T3 B_reg_ = 0.58; T2-T3 B_neg_ = 3.98). Infant behavioral temperament (regulation and negative emotionality) and maternal depressive symptoms had significant within-person associations for each time point (Figs. 2, 3).

#### Infant behavioral temperament is associated with later taxa richness.

Infant behavioral temperament and infant Chao1 LKT were associated such that higher levels of regulation at T1 and negative emotionality at T1 were associated with less diversity at T2 (B_reg_ = −30.11; B_neg_ = −0.17) and lower levels of regulation and higher levels of negative emotionality were associated with less diversity at T3 (B_reg_ = 76.37; B_neg_ = −1.06; see Figs. 2, 3). Only infant Chao1 LKT T1 was associated with negative emotionality at T2 (B_neg_ = −0.37; all other CIs included zero).

#### Maternal taxa richness and its association with maternal depressive symptoms.

Only two paths between maternal depression and maternal taxa richness (Chao1 LKT) were significant in the negative emotionality model (maternal depression T1 and maternal Chao1 LKT T2 B_neg_ =−0.10, maternal Chao1 LKT T1 and maternal depression T3 B_neg_ = 2.93; all CIs included zero in the regulation model). Here, it is important to note that the same within-subjects paths for mothers between the microbiome and depressive symptoms can differ between the two models which differ only in the infant temperament outcome. This may in part be due to the paths between infant regulation and maternal depressive symptoms or infant regulation and the maternal microbiome accounting for some of the variance. In the regulation model, the significant cross-lagged path from infant regulation (T2) to the maternal microbiome (T3) accounts for a substantial portion of the variance in the T3 maternal microbiome, thereby reducing the magnitude of the direct autoregressive path from the T2 maternal microbiome. This effect is not observed in the negative emotionality model, where the corresponding cross-lagged path is non-significant, resulting in a stronger apparent stability for the maternal microbiome in that model.

### Between-individual associations between gut microbiota and behavior

#### Early maternal depressive symptoms are not associated with later temperament.

Infant temperament at T2 and maternal depressive symptoms at T3 were associated, whereby higher levels of infant regulation and negative emotionality were associated with higher levels of maternal depressive symptoms (B_reg_ = 19.15; B_neg_ = 0.34).

#### Maternal taxa richness and its associations with infant taxa richness.

Associations between infant Chao1 LKT and maternal Chao1 LKT were only found for the negative emotionality model such that infant Chao1 LKT was positively associated with maternal Chao1 LKT at T2 (B_neg_ = 0.01). Maternal Chao1 LKT at T1 was negatively associated with infant Chao1 LKT at T2 (B_neg_ = −0.02), whereas maternal Chao1 LKT at T2 was positively associated with infant Chao1 LKT at T3 (B_neg_ = 0.56).

#### Infant taxa richness is negatively associated with maternal depressive symptoms.

Infant Chao1 LKT at T1 (B_reg_ = −0.11) and T2 (B_neg_ = −0.61) were negatively associated with maternal depressive symptoms at T2 and T3, respectively. Maternal depressive symptoms at T2 were positively associated with infant Chao1 LKT at T3 (B_neg_ = 0.24).

#### Maternal taxa richness is associated with infant temperament.

Lower regulation and higher negative emotionality at T1 were associated with lower maternal Chao1 LKT at T2 (B_reg_ = 90.77; B_neg_ = −0.04). Infant regulation at T2 was negatively associated with maternal Chao1 LKT at T3 (B_reg_ = −69.43) and maternal Chao1 LKT at T2 was positively associated with infant regulation at T2 (B_reg_ = 0.54).

### Individual microbes in the infant’s gut, but not the mother’s gut, were associated with infant temperament and maternal depression

**Fig. 4:**
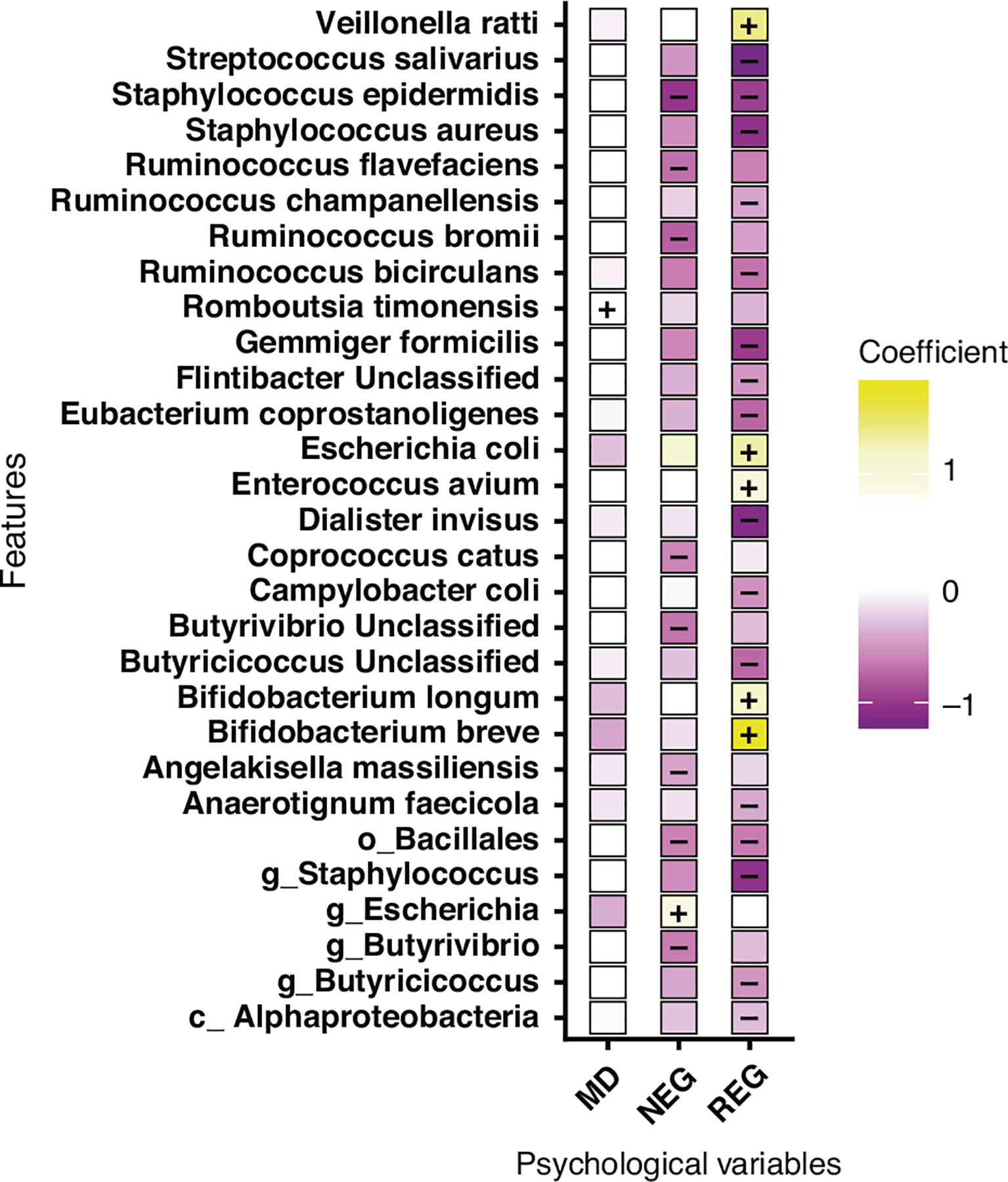
Associations between infant taxa, infant temperament, and maternal depressive symptoms (*N* = 121). Microbiome Multivariate Association with Linear Models (Maaslin2) was used to identify potential microbial biomarkers of infant temperament and maternal depression. Analyses were conducted separately for infants’ and mothers’ microbial taxa and features. For these models, infant negative affectivity, infant regulation, maternal depression, age in months at assessment, and principal components (PC1:PC4) were entered as fixed effects and subject was entered as a random effect. Only taxa identified as significant at *q* < 0.05 are shown. g- identified at the genus level, LKT Last known taxa, MD maternal depressive symptoms, NEG negative affectivity, REG regulation.

Several infant microorganisms including Butyrivibrio (genus), Bacillales (order), *Staphylococcus epidermidis*, *Ruminococcus flavefaciens, Coprococcus catus*, Escherichia (genus), *Angelakisella massiliensis, and Ruminococcus bromii* were associated with infant negative emotionality. Similarly, Staphylococcus (genus), *Dialister invisus, Eubacterium coprostanoligenes, Campylobacter coli, Bifidobacterium breve, Staphylococcus aureus*, Bacillales (order), *Streptococcus salivarius*, *Veillonella ratti*, *Gemmiger formicilis*, *Staphylococcus epidermidis*, Butyricicoccus (genus), *Ruminococcus champanellensis*, *Anaerotignum faecicola*, Flintibacter (genus), Alphaproteobacteria (class), *Bifidobacterium longum*, *Escherichia coli*, Butyricicoccus (genus), *Ruminococcus bicirculans*, and *Enterococcus avium* were associated with regulation (see Fig. 4 and Supplementary Table S10). In addition, infant *Romboutsia timonensis* was positively associated with maternal depressive symptoms. None of the maternal microorganisms were significantly associated with infant behavioral temperament or maternal depression. For the associations with functional terms see Supplementary Tables S11, 12.

## DISCUSSION

The present study examined the within-person and bidirectional relationship between maternal and infant gut microbiota and its effects on maternal and infant behavior. Consistent with prior research, our results show that the infant gut microbiome undergoes substantial group-level development during the first year of life as it increases in its compositional and functional diversity.^1,58–61^ At the individual level, both maternal and infant gut microbiota diversity show a degree of consistency across time points with the maternal microbiome at T1 being a better predictor of T2 diversity levels than the infant microbiome. A series of infant gut microbes and functional terms were of relevance to infant temperament. However, there were no maternal taxa or functional terms related to maternal depression. These findings provide insights into the early development of the gut microbiome-brain axis and its associations with behavioral outcomes relevant to mental health.

### Infants, but not postpartum mothers, exhibit group-level changes in taxa diversity across the first year of the infant’s life

In line with previous work, infant gut microbiome richness increases with time.^1,58–62^ Prior work has found that infant diet is the most important contributor to early microbiota composition.^62^ These developmental changes were also evident at the genus level, with newborns displaying higher levels of *Bifidobacteria* and *Escherichia*, whereas at 14-month-olds showed higher levels of *Bacteroides* and *Faecalibacterium and show a composition remarkably similar to their mother*’*s profile*. The maternal gut microbiome was found to be more consistent than the infant gut microbiome from T1 to T2 but not T2 to T3.^6^ Despite the great amount of change brought on by a recent delivery of an infant (e.g., antibiotics, Pitocin) and by care practices (e.g., breastfeeding, disruptions in sleep, etc.), the maternal gut microbiome remains relatively unperturbed.^63^ Examination of functional terms contrasts with the taxa findings such that infants have higher mean levels of antibiotic resistance and virulence factor genes than their mothers. This early compositional profile is likely due to the high levels of competition and selective pressures (e.g., antibiotic exposure, infections, etc.) that microbes face^64^ during the first year of life.

### Within-individual associations between taxa diversity and behavior

In contrast to prior work,^18^ early infant taxa diversity was not associated with later behavioral regulation among infants in the current study. Only taxa diversity in the newborn period was associated with negative emotionality, such that greater newborn diversity was associated with less negative emotionality at 7 months. Additionally, our study revealed associations between early temperament and later gut microbiota diversity with greater negative emotionality and regulation in the newborn period associated with less infant gut microbiome taxa diversity at 7 months. In contrast, results also showed that greater negative emotionality and less regulation at 7 months were associated with less diversity at 14 months. These results point to nonlinear trajectories of growth. Further work will be needed to understand how environmental factors such as birth methods and feeding methods contribute to such growth trajectories.

Using multiple measures of both gut microbiome composition and behavior across time points allowed us to identify longitudinal associations while controlling for potential association effects driven by behavioral associations alone, which was not controlled for in previous research, thus reflecting strength in our approach. Finding that behavioral temperament is associated with later gut microbial diversity supports the hypothesis that certain predispositions and temperament traits contribute to how an infant interacts with the world, which in turn affects how colonization with microbiomes unfolds during early human development.^65^ More specifically, it appears that newborn infants displaying high levels of both negative emotionality and regulation are the ones that go on to develop less diverse microbiomes. This pattern may be particular to newborn behavioral temperament, as high levels of negative emotionality and low levels of regulation at age 7 months were associated with less diverse microbiomes at 14 months. Overall, this suggests that newborns with greater negative affectivity have less diverse microbiomes. Future work should investigate why such patterns emerge, exploring the possibility that infants with greater negative affectivity have reduced exposure to novel social and physical environments.

Another possibility for why infant gut microbiota was not associated with later behavioral temperament is that a type of microbiota profile leads to a host of behaviors not directly assessed in the present analyses like infant fussiness, lethargy, or sickness.^66^ Here, it is important to note that supplemental analyses assessed if subcomponents of temperament were related to both maternal and infant gut microbiome diversity. In these analyses, no robust and clear patterns were observed (see Supplementary Fig. S8). Furthermore, exploratory analyses were conducted to test the associations between infant health symptoms assessed using maternal report at age 7 months, gut microbiota composition, and psychological outcomes; and significant associations were found (see Supplementary Figs. S9, 10). These exploratory associations shed light on the interconnection between somatic and psychological health. More systematic work is needed to understand these associations.

In contrast to previous studies, none of the individual maternal taxa were significantly related to maternal depression.^67,68^ In addition, none of the maternal taxa were of relevance to infant temperament, and the taxa diversity results were inconsistent between the two temperament models. One possibility as to why the present study was unable to identify candidate taxa and functional terms is that there is heterogeneity in postpartum depression, such that some mothers had clinical depression before their pregnancy, and for som,e the symptoms of depression start shortly after giving birth. Therefore, one possibility is that the microorganisms underlying depression change across the pre-pregnancy, prenatal, and postnatal periods. Another possibility is that a single microorganism is unable to explain the differences in behavior and it is the interactions between organisms, their metabolites, or the greater community that matter.

In line with previous work, individual infant taxa and functional terms were associated with infant temperament.^18,21^ Several of the infant taxa linked to behavioral temperament, such as *Staphylococcus* and *Escherichia coli* have been implicated in disease models (skin infection, respiratory infection, and gastrointestinal distress).^69,70^ The model identified several common genus-level bacteria that have been linked to positive mental health outcomes in adults, such as *Bifidobacterium*.^11^ In addition, *Veillonella ratti* was positively associated with regulatory behaviors, and in prior work has been shown to be elevated in children with autism.^71^

By examining shared genetic materials across the microbiome with functional term analyses, we were able to overcome missed identifications at the taxa level and examine potential underlying functions in our analysis. Several of the candidate antibiotic resistance genes identified in infants’ guts as being of relevance to behavioral temperament (acrE, acrS) were known targets of the antibiotic cephalosporin.^72^ Cephalosporin is a glutamate transporter subtype 1 enhancer and has been shown to reduce brain-derived neurotrophic factor levels in the hippocampus and enhance depressive symptoms.^45,73,74^ Previous studies suggest a positive link between antidepressant use and the transfer of antibiotic-resistance genes across bacteria.^75^ These insights highlight the advantages of the shotgun sequencing methods used in the current study over the more limited 16S rDNA sequencing methods.^1^ It also extends previous work, which has found associations (both positive and negative) between antibiotic administration and depressive symptoms.^36,45^

### Between-individual associations between gut microbiota and behavior

Our analysis identified a single, exploratory association between the relative abundance of an infant microbe, Romboutsia timonensis, and maternal depressive symptoms. This finding should be interpreted with extreme caution. First, this was an unexpected finding for which we had no prior hypothesis, and it emerged from a large number of statistical comparisons, increasing the likelihood of a Type I error. Second, the biological plausibility of a single, low-abundance infant gut microbe functionally influencing maternal neurobiology is very low. As noted in Supplementary Table S15, *Romboutsia timonensis* is not a dominant member of the infant gut community, especially early in infancy. Therefore, rather than representing a direct functional link, it is more probable that this statistical association is spurious or reflects an unmeasured confounding variable related to the shared dyadic environment. For example, factors such as shared diet, maternal stress (which can independently influence both maternal mood and the infant’s gut colonization), or other environmental exposures could give rise to such a correlation without any direct causal link. Future studies with integrated metabolomic data and carefully controlled experimental designs would be required to determine if this association is reproducible and has any biological relevance.

### Covariates and limitations of the present study

The present study was also able to examine gut-brain associations while controlling for several important factors through the use of dimension reduction. This technique is helpful to test for associations that go above and beyond known factors that manipulate the gut microbiota. However, it also does not allow for an in-depth examination of important moderating or mediating factors like infant diet and antibiotic administration. In line with previous work, breastfeeding was negatively associated with taxa diversity whereas formula feeding was positively associated with taxa diversity.^76^ Moreover, formula feeding was positively associated with regulation behaviors at T3, hinting at a possible moderation or mediation. For antibiotics, the antibiotic administration during labor, but not subsequent administration of antibiotics to the child was positively associated with gut microbiota taxa diversity at T1 and negatively associated with taxa diversity at T3. There was also a positive association between antibiotic administration during labor and negative affectivity at T3, again hinting at possible mediating or moderating effects. Future work should continue to disentangle these complex associations. This study is also limited by the absence of metabolomic data, which restricts our ability to link gut microbial composition to functional metabolic outputs (e.g., amino acids and short-chain fatty acids), which may more directly interface with central nervous system functioning and impact behavior. It would be interesting for future work to assess if and how the metabolome mediates the association between gut microbiota and behavior.^77^

The present study has several strengths including the use of rigorous methods, such as the use of shotgun-metagenomic sequencing, and the longitudinal assessment of both maternal and infant gut microbiomes. Nonetheless, it is important to contextualize the findings within their limitations. One limitation is the reliance on parental report data for both maternal depressive symptoms and infant behavioral temperament outcomes. Previous work has shown that parental reports of infant behaviors are in part influenced by the parent’s mental health.^78^ We encourage future work to use both parent reports and observational measures to better understand how reported and observed behaviors are similarly and differentially associated with the gut microbiota. The use of parent reports also has strengths in that it helps to make this study comparable to many of the other gut microbiome studies that have used the same assessment of behavioral temperament.^20,79^ Relatedly, the Edinburgh Postnatal Depressive Scale measures both depressive and anxiety symptoms.^54^ Therefore, future work may benefit from including additional mental health questionnaires and clinical assessments to disentangle if microbe-mental health pathways are specific to depression or anxiety. The present study is also limited in that it did not collect stool samples and behavioral data from the mothers during their pregnancy. These data would help to characterize the changes within the mother during the prenatal to postnatal transition period and this time period may be of particular relevance for the vertical transition of microbes to the infant. A further limitation is the freezing of stool samples occurred within 24 h. This protocol has been used in other published work^21^ and helped ease the study burden for mothers and infants participating. Prior work has shown mixed results in terms of the effects of delaying freezing time such that some studies point to changes in microbial communities happening after 48 h at room temperature, whereas others point to marked consistency.^80–83^ The time between collection and freezing showed little effect on the gut microbiome compositions of infants and mothers (see Supplementary Figs. S1–6). Stool samples are limited in their generalizability to the entire intestinal tract.^84^ Methods such as colonoscopies, biopsies of the intestinal tract, or ingestible devices may provide a more comprehensive assessment of the gut microbiota.^85^ However, stool samples remain the preferred biospecimen to use as it is non-invasive and relatively easy to collect from pediatric and adult participants. There are many other important collection sites for the microbiome, including the skin, vagina, and mouth, which were not assessed. Future work would thus benefit from examining the link between the gut microbiome, other sites’ microbiomes, and maternal and infant mental health outcomes.

In conclusion, the present study provides important insights into the dyadic, bidirectional relation between maternal and infant gut microbiota and its effects on maternal and infant affect and behavior. Major contributions of the present study include: (a) identifying several infant taxa linked to behavioral temperament, and (b) characterizing the trajectories of microbial composition in mothers and infants across the first postnatal year. Overall, the present findings shed new light on how gut biology, family systems, and behavior interact during early human development.

## Supplementary Material

Supplementary Table S6

Supplementary Table S7

Supplementary Table S8

Supplementary Table S9

Supplementary Table S11

Supplementary Table S10

Supplementary Table S2

Supplementary Table S12

Supplementary Table S15

Supplementary Table S3

Supplementary Table S4

Supplementary Table S5

Supplementary Materials

**Supplementary information** The online version contains supplementary material available at https://doi.org/10.1038/s41390-025-04630-9.

## Figures and Tables

**Table 1.** Socio-demographic and health information for the mother-infant dyads (*N* = 121)

Socio-demographic information	Mean | *n* (*SD* | %)
**Family**	
Income, *n*	
Less than $15,000	8 (6.7%)
$15,001 to $30,000	21 (17.5%)
$30,001 to $45,000	16 (13.3%)
$45,001 to $60,000	10 (8.3%)
$60,001 to $75,000	6 (5.0%)
$75,001 to $90,000	11 (9.2%)
$90,001 to $110,000	15 (12.5%)
$110,001 to $125,000	6 (5.0%)
$125,001 to $175,000	15 (12.5%)
$175,001 to $225,000	7 (5.8%)
$225,001 to $275,000	1 (0.8%)
$275,001+	4 (3.3%)
Maternal education	
Some High School	3 (2.5%)
High School Diploma/GED	18 (14.9%)
Some College/Associates	24 (19.8%)
Bachelor's degree	29 (24.0%)
Graduate degree	47 (38.8%)
Number of siblings at the time of birth	1.12 (1.07)
**Mother**	
Maternal age at birth	31.75 (4.59)
Race	
White	104 (86.0%)
Black	12 (9.9%)
East Asian	2 (1.7%)
South Asian	3 (2.5%)
Pacific Islander	2 (1.7%)
Other	4 (3.3%)
Antibiotic administration during labor	74 (61.7%)
Antibiotic administration after birth	23 (19.0%)
BMI at study enrollment	27.54 (6.17)
**Infant**	
Age at T1, days	25.27 (9.13)
Age at T2, months	7.69 (0.59)
Age at T3, months	14.56 (0.64)
Female sex assigned at birth	49 (40.5%)
Race, *n*	
White	106 (87.6%)
Black	20 (16.5%)
East Asian	3 (2.5%)
South Asian	4 (3.3%)
Pacific Islander	3 (2.5%)
Other	6 (4.9%)
Vaginal delivery, *n*	87 (71.9%)
Birthweight, grams	3445.16 (446.43)
Gestational age, months	39.57 (1.15)
Apgar Score	8.17 (1.11)
Antibiotic administration after birth	22 (18.2%)

Participants were allowed to select multiple identities. Therefore, percentages may exceed 100%. *T1*- Time 1, *T2* – Time 2, *T3* – Time 3, *BMI* – Body Mass Index.

## Data Availability

The raw sequencing reads used in this study will be made publicly available through the National Center for Biotechnology Information (NCBI). Some of the other data used in the manuscript are identifiable and may also contain sensitive health information and is therefore only available upon request. Requests should be sent to the corresponding authors. Data sharing may be possible with additional IRB review and data use agreements.

## IMPACT:

- How the gut microbiome influences maternal mental health and infant behavior remains poorly understood.
- We measured mothers’ and infants’ gut microbiota composition and behavior across the first year of the infant’s life.
- Individual taxa from the infant, but not the maternal, gut were associated with infant behavioral temperament.
- Our findings provide evidence for complex bidirectional gut-behavior associations between mothers and infants.
