# Supplementary material for: Bidirectional relations between the maternal and infant gut microbiome and behavior
Source: Pediatr Res. Author manuscript; Available in PMC 2026 Jan 3. (PMC12758567; doi:10.1038/s41390-025-04630-9)
Supplement: Supplementary Materials [file NIHMS2131853-supplement-Supplementary_Materials.pdf]

Bidirectional relations between the maternal and infant gut microbiome and behavior

Supplementary Materials

Supplementary methods

Stool collection and shotgun metagenomic analysis.

Parents were instructed to collect their stool and their infant stool samples at home using a sterile biospecimen container and infant diapers respectively. See [1] for a previous study using this protocol. Stool samples were then transferred to cryovials containing a 20% Glycerol and 80% Phosphate-Buffered Saline solution and stored at -80°C (*Mean time before freezing* = 9.79 hours, *SD* = 8.71 hours). Samples were processed and sequenced at the National Cancer Institute (NCI). Automated DNA extraction was performed with the MagAttract PowerMicrobiome DNA/RNA kit (Qiagen, Cat No./ID: 27500-4-EP) with Qubit™ quantification following the manufacturer's instructions. Samples that fell below quality control standards for DNA concentrations were removed from further analyses. Library preparation and sequencing was completed using the Illumina Nextera DNA Flex Library Prep and Illumina NovaSeq 6000 sequencing platform, respectively.

The shotgun sequencing output was analyzed using a series of pipelines and functions in the R language developed in-house and publicly available on Github under the package name Just Another Microbiology System (JAMS), found at [2]. Details of this method can be found in supplementary materials and in other manuscripts [3-5]. JAMS was implemented on the high-performance computer, Biowulf at the National Institutes of Health. Downstream analysis was performed locally on a secure computer. The paired-end sequencing reads were (1) quality trimmed using Trimmomatic [6], (2) aligned to the human genome using Bowtie2 and host DNA was subsequently removed [7], and (3) assembled into contigs using Megahit [8]. Contigs were then assigned taxa using k-mer analysis with kraken2 [9], using a custom-built database containing all genomes of all Bacteria, Archaea, Fungi, Viruses and Protozoa deposited in NCBI GenBank.

Contigs were also annotated, *ab initio,* using Prokka [10], yielding the predicted proteome for the metagenomic sample. Sequencing reads were then aligned back to assembled contigs to compute base pair counts for each contig. The total base pair count for each last known taxon (LKT) – deepest taxonomic level up to species confidently classified using kraken2, was computed as the number of bases covering all contigs classified for each LKT. Note, Kraken2 was chosen for its comprehensive, fast, and accurate taxonomic profiling. However, this method has been known to have a few false positives [11,12] . The predicted proteome of each metagenomic sample (translated genes found within contigs) was further functionally classified into Gene Ontology Terms [13], virulence factors (virulence factor data base [VFDBs]; [14]), and antibiotic resistance genes [15-19]. Hits with <75% identity and/or <75% query coverage were discarded.

The relative abundance, in parts per million (PPM) of each feature was used in subsequent analyses. This is obtained by dividing the number of bases covering a feature by the total number of bases sequenced for that analysis in a sample multiplied by 10^6. For alpha-diversity analyses, alpha-diversity measures of Chao1, Shannon Diversity Index, and Faith's phylogenetic diversity (see supplementary Figures 1-6 for correlation analyses using Shannon and Faith's PD) were obtained using the Vegan and picante packages in R.

Behavioral Temperament

Two temperament dimensions were computed summarizing information from various sub-scales: (1) negative emotionality (example sub-scales: fear and sadness [referred to as negative affectivity in the ECBQ]; T1: $M = 2.57$; $SD = .88$; T2: $M = 3.19$, $SD = .77$; T3: $M = 2.89$, $SD = .51$) and (2) regulation (example sub-scales: low intensity pleasure, inhibitory control, and soothability [referred to as effortful control in the ECBQ]; T1: $M = 3.97$; $SD = .53$; T2: $M = 5.12$, $SD = .57$; T3: $M = 4.42$, $SD = .55$) [20]. Surgency (example sub-scales: approach, vocal

reactivity, smiling and laughing) and all of the subscale scores were also computed and included in analyses in the supplementary materials.

Covariate inclusion in models.

Principal component analysis (PCA; psych package in R) was used as a dimension reduction technique to allow for the maximal inclusion of covariates in subsequent analyses (see supplemental materials). Sixteen variables were entered into the PCA analysis: maternal age at birth, child sex assigned at birth (1 = Female; 0 = Male), maternal education (1 = some High School; 5 = Graduate Degree), child's race (1 = Non-white; 0 = White), delivery method (1 = c-section; 0 = vaginal), gestational age, birthweight, maternal BMI, antibiotic administration during labor (1 = yes; 0 = no), antibiotic administration tomother after birth (1 = yes; 0 = no), administered to child after birth (1 = yes; 0 = no), breastfeeding at T2 (1 = yes; 0 = no), Formula feeding at T2 (1 = yes; 0 = no), maternal fruit intake, maternal vegeTable Sintake, and maternal high sugar or high-fat food intake. Antibiotic administration was assessed using self-report. If the parent indicated any antibiotic use at T1, T2, or T3, antibiotic administration after birth was coded as yes. Infant diet was assessed through parent report questionnaires asking if any breastmilk or formula had been fed to the infant within the last week (note, these are not mutually exclusive questions). Maternal diet was assessed using a food frequency questionnaire [21]. The average amounts for each of the food categories were used. Non-parametric missing value imputation using random forest (missForest package in R) was used to impute scores for individuals who had data for some but not all of the covariates of interest [22]. Four factors were retained, as suggested by parallel analysis, and no rotation was applied in the computation of the principal components in order to reduce collinearity between components (for factor loading see **Supplementary Table S1**).

Supplementary results

Sex differences

Sex differences were examined using Spearman's rank correlations (see Supplementary Figures 1-3). Briefly females, compared to males, had greater gut microbiota taxa diversity at T1 (Faith's PD LKT) and T2 (Chao1 LKT, Shannon LKT, and Faith's PD LKT). In addition, females had lower ratings of negative affectivity at T3, compared to males.

Summary of group level changes using Shannon and Faith's Phylogenetic Diversity (PD) taxa diversity metrics.

Shannon. When assessing group-level changes in the maternal and infant for Shannon LKT, there was a significant main effect of individuals and time. However, this was qualified by an individual-by-time interaction ($B = -.82$, $SE = .06$, $t = -13.32$, $p < .001$). Post hoc analyses revealed that infants' Shannon diversity increased over time (all *p-values* < .001) but maternal Shannon LKT levels were not significantly different over time ($p = 1.00$). Moreover, at all-time points, infants had significantly lower Shannon LKT levels than their mothers (all *p*-values < .001; see Figure 11).

Faith's Phylogenetic Diversity (PD). When assessing group-level changes in the maternal and infant for Faith PD LKT, there was a significant main effect of individuals and time. However, this was qualified by an individual-by-time interaction ($B = -1.40$, $SE = .13$, $t = -10.58$, $p < .001$). Post hoc analyses revealed that infants' Faith PD LKT increased over time (all *p-values* < .001) but maternal Faith PD LKT levels were not significantly different over time ($p = 1.00$). Moreover, at all-time points, infants had significantly lower Faith PD LKT levels than their mothers (all *p*-values < .001; see Figure 12).

Summary of results using functional group diversity.

Group level changes across the first year of life. When assessing group-level changes in the maternal and infant Chao1 antibiotic resistance genes, there was a significant effect of individuals such that infants had greater levels of antibiotic resistance genes than mothers (B = 12.77, SE = 4.13, t = 3.10, p = .002). However, there was no main effect of time or individual-by-time interaction (all p-values > .225; see Supplementary Figure 13).

When assessing group-level changes in the maternal and infant Chao1 virulence factors, there was a significant effect of individuals such that infants had greater levels of virulence factors than mothers (B = 38.69, SE = 7.43, t = 5.21, p < .001). However, there was no main effect of time or individual-by-time interaction (all p-values > .82; see Supplementary Figure 14).

When assessing group-level changes in the maternal and infant Chao1 GO Terms, there was a significant effect of time (B = 34.48, SE = 15.18, t = 2.27, p = 0.024). Post hoc analyses revealed that there was a significant increase in GO Terms from T1 to T2 (p < .001), but not between T2 and T3 or T1 and T3 (*p-values* > .324; see Supplementary Figure 15). There were no other significant main effects or interaction effects between individuals and time.

Within-individual and between-individual associations between gut microbiota and behavior. Six separate Markov Chain Monte Carlo (MCMC) models were conducted to assess the relation between mother and infant gut microbiome (alpha diversity of functional terms: Gene Ontology (GO) terms, virulence factors, and antibiotic resistance genes and mental health outcomes (depression and behavioral temperament [negative emotionality and regulation separately]). For the full results see Supplementary Tables 2-9.

Figures

Supplementary Figure S1

Spearman's rank correlations between clinical covariates and study variables for infants at time 1.

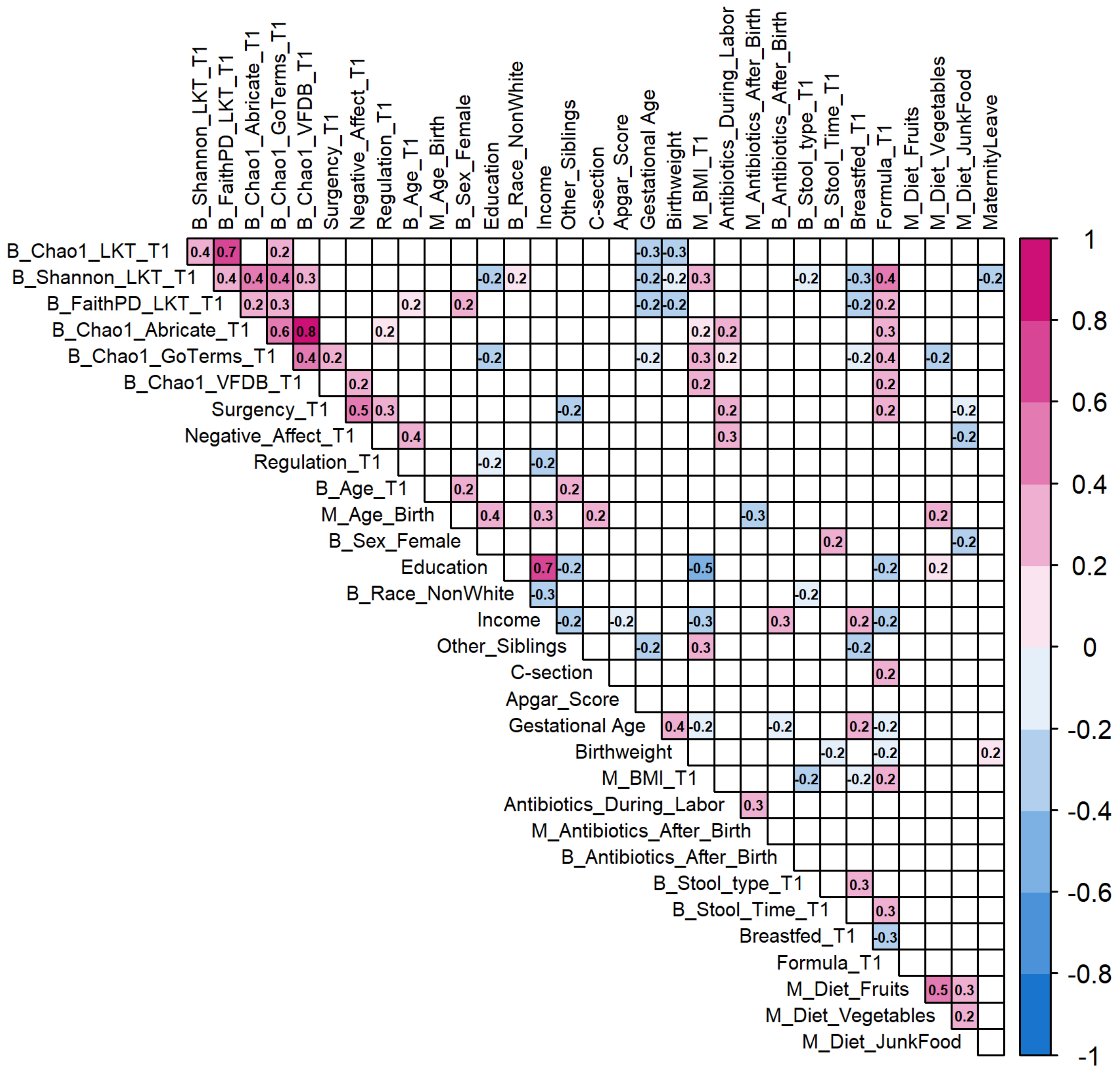

Note. Blank cells represent nonsignificant associations ($p > .05$). Abbreviations: Abricate – antibiotic resistance, B - Baby, BMI - Body Mass Index, LKT - Last Known Taxa, M -Mother, PD – Phylogenetic Diversity, T - Time, VFDB - Virulence Factor Database.

Supplementary Figure S2

Spearman's rank correlations between clinical covariates and study variables for infants at time 2.

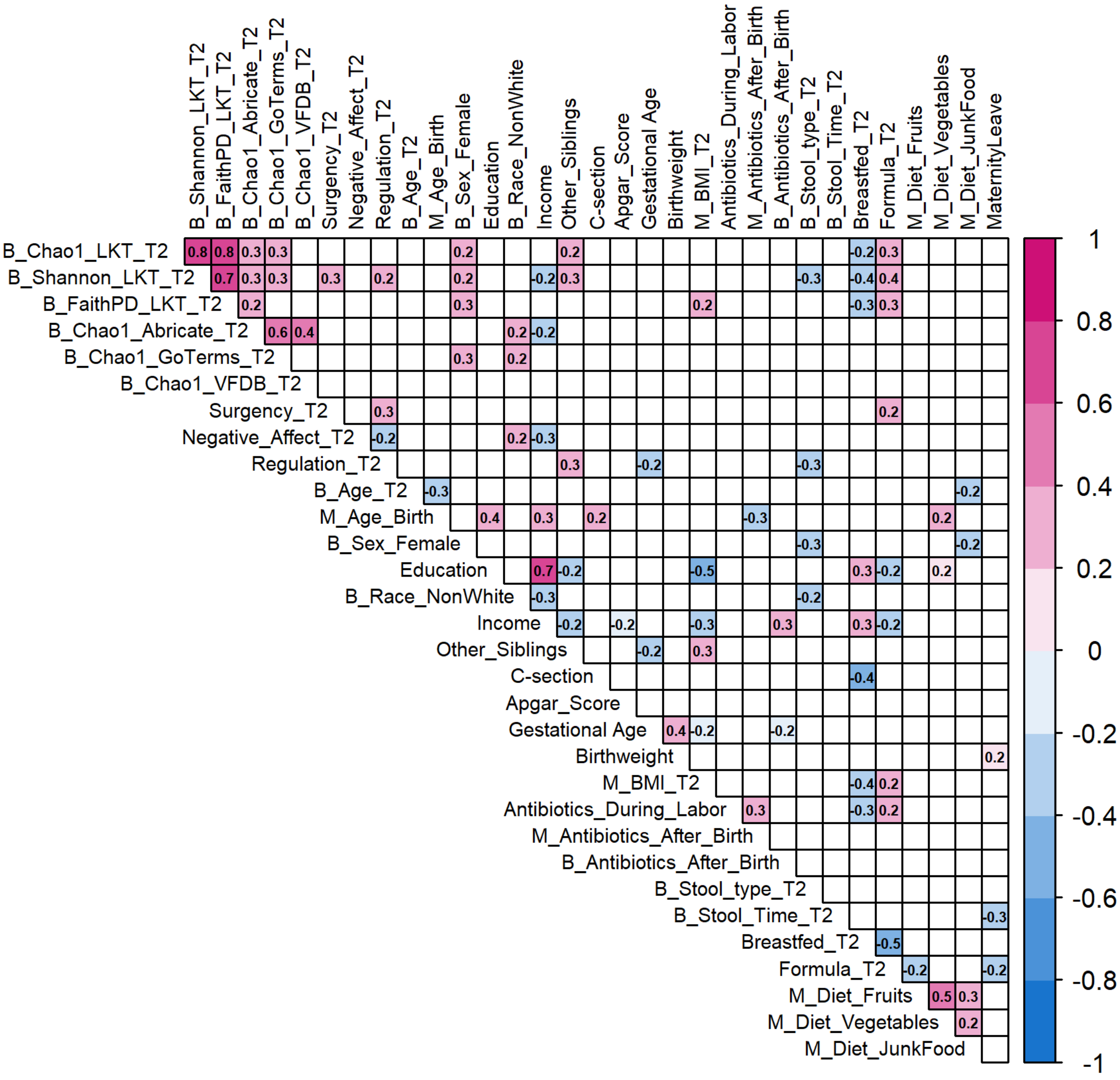

Note. Blank cells represent nonsignificant associations ($p > .05$). Abbreviations: Abricate – antibiotic resistance, B - Baby, BMI - Body Mass Index, LKT - Last Known Taxa, M - Mother, PD – Phylogenetic Diversity, T - Time, VFDB - Virulence Factor Database.

Supplementary Figure S3

Spearman's rank correlations between clinical covariates and study variables for infants at time 3.

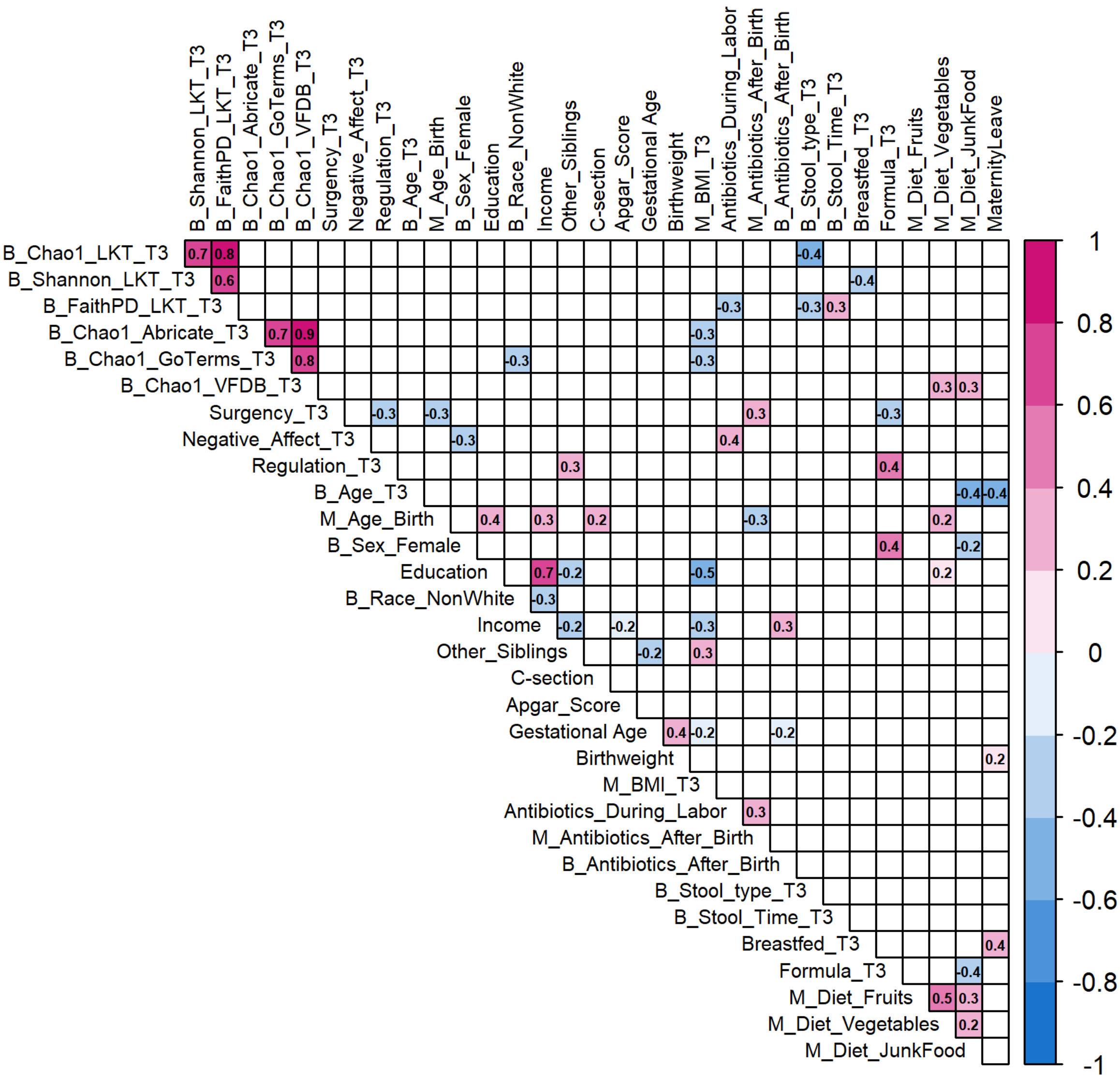

Note. Blank cells represent nonsignificant associations ($p >$ .05). Abbreviations: Abricate – antibiotic resistance, B - Baby, BMI - Body Mass Index, LKT - Last Known Taxa, M - Mother, PD – Phylogenetic Diversity, T - Time, VFDB - Virulence Factor Database.

Supplementary Figure S4

Spearman's rank correlations between clinical covariates and study variables for mothers at time 1.

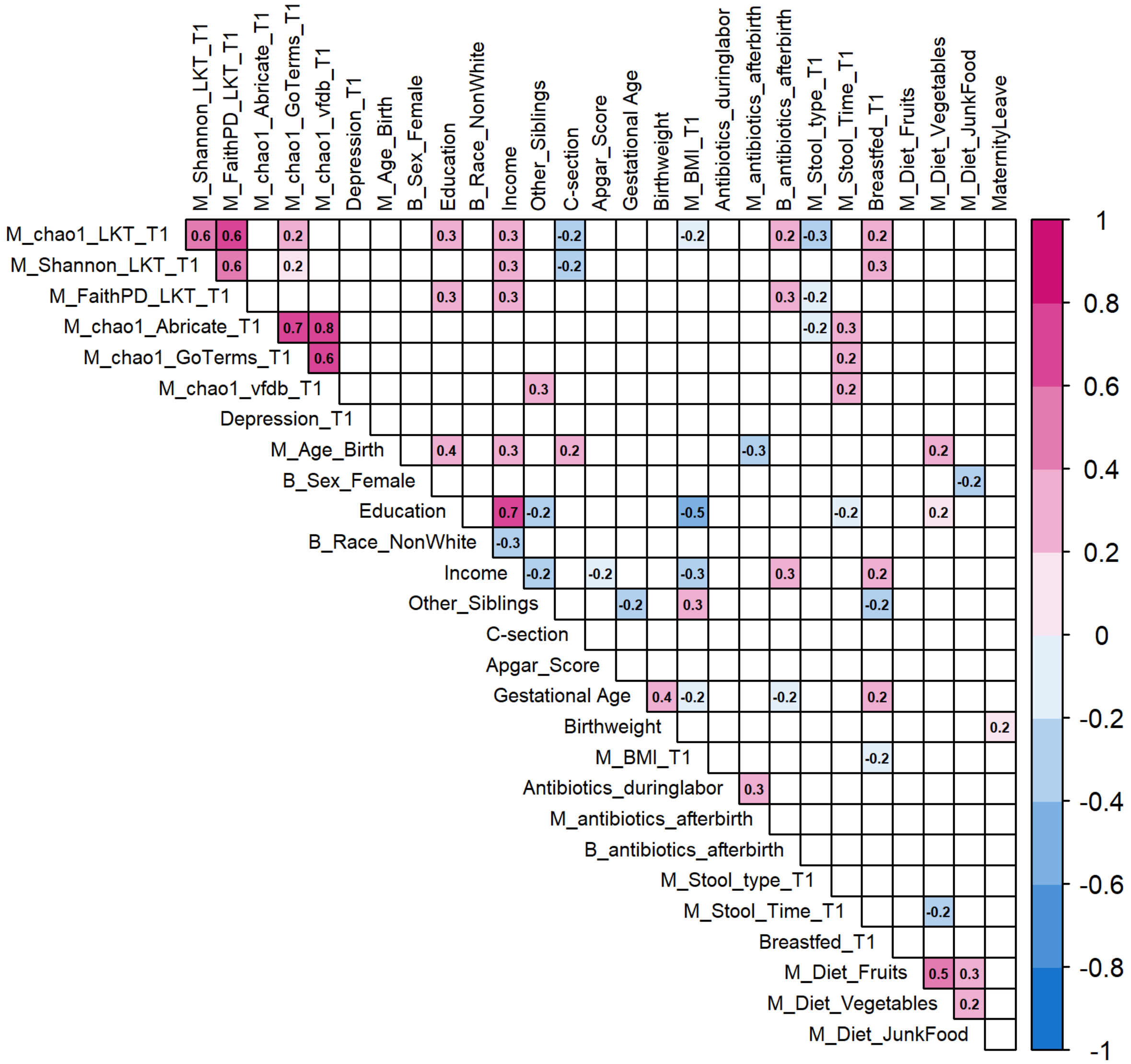

Note. Blank cells represent nonsignificant associations ($p >$ .05). Abbreviations: Abricate – antibiotic resistance, B - Baby, BMI - Body Mass Index, LKT - Last Known Taxa, M - Mother, PD – Phylogenetic Diversity, T - Time, VFDB - Virulence Factor Database.

Supplementary Figure S5

Spearman's rank correlations between clinical covariates and study variables for mothers at time 2.

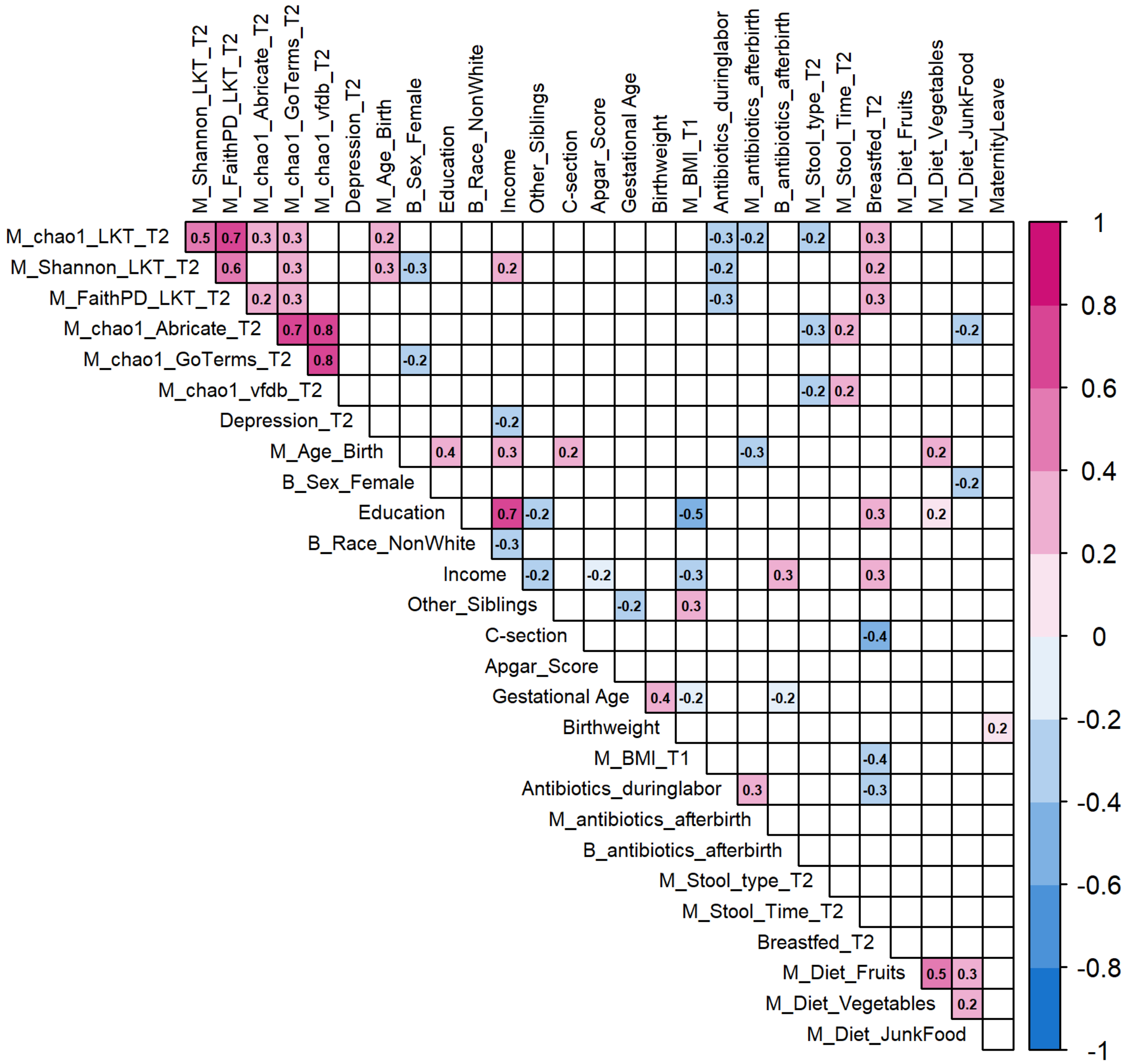

Note. Blank cells represent nonsignificant associations ($p >$ .05). Abbreviations: Abricate – antibiotic resistance, B - Baby, BMI - Body Mass Index, LKT - Last Known Taxa, M - Mother, PD – Phylogenetic Diversity, T - Time, VFDB - Virulence Factor Database.

Supplementary Figure S6

Spearman's rank correlations between clinical covariates and study variables for mothers at time 3.

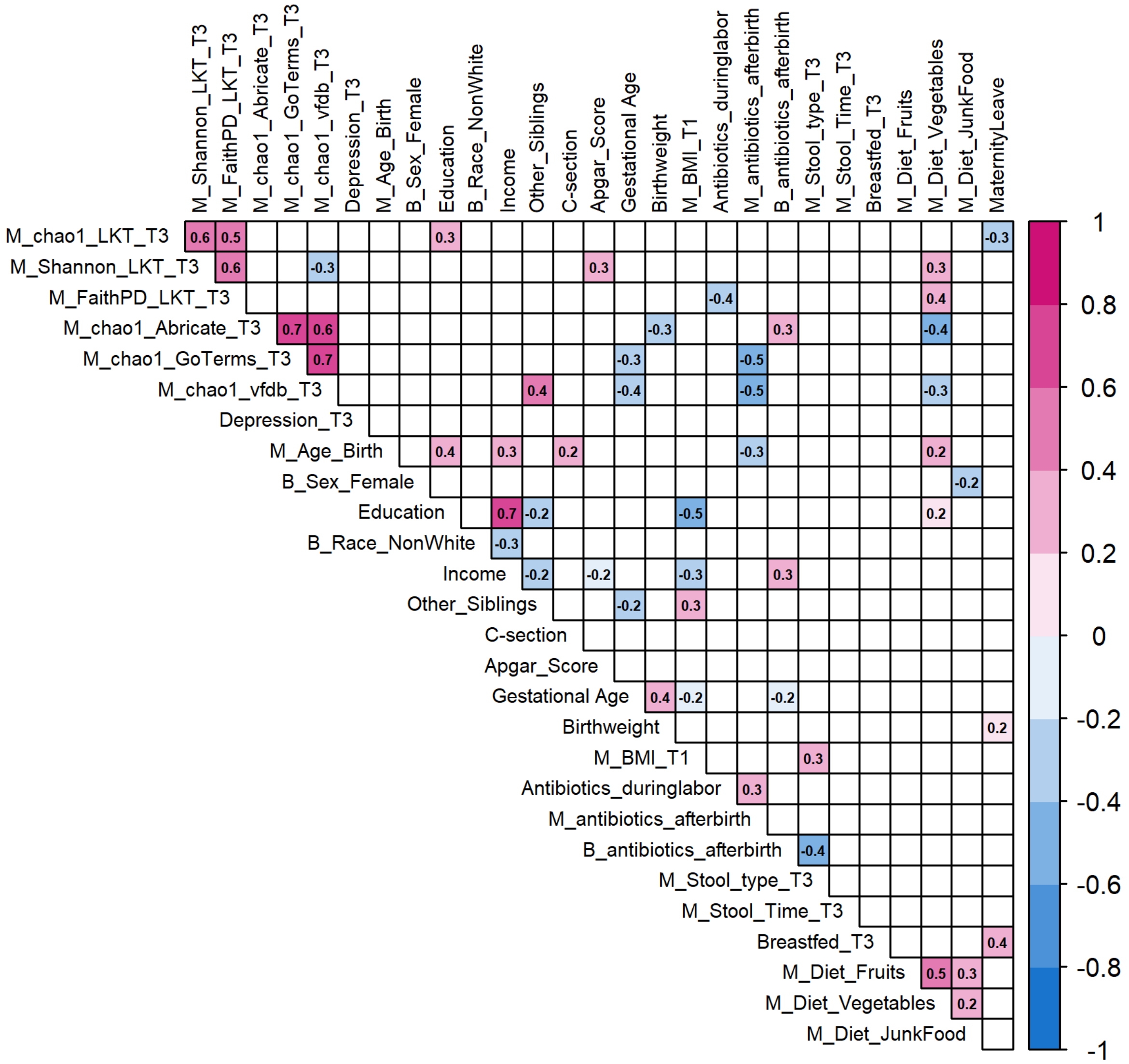

Note. Blank cells represent nonsignificant associations ($p > .05$). Abbreviations: Abricate – antibiotic resistance, B - Baby, BMI - Body Mass Index, LKT - Last Known Taxa, M - Mother, PD – Phylogenetic Diversity, T - Time, VFDB - Virulence Factor Database.

Supplementary Figure S7

Spearman's rank correlations between maternal and infant gut microbiomes at each time point.

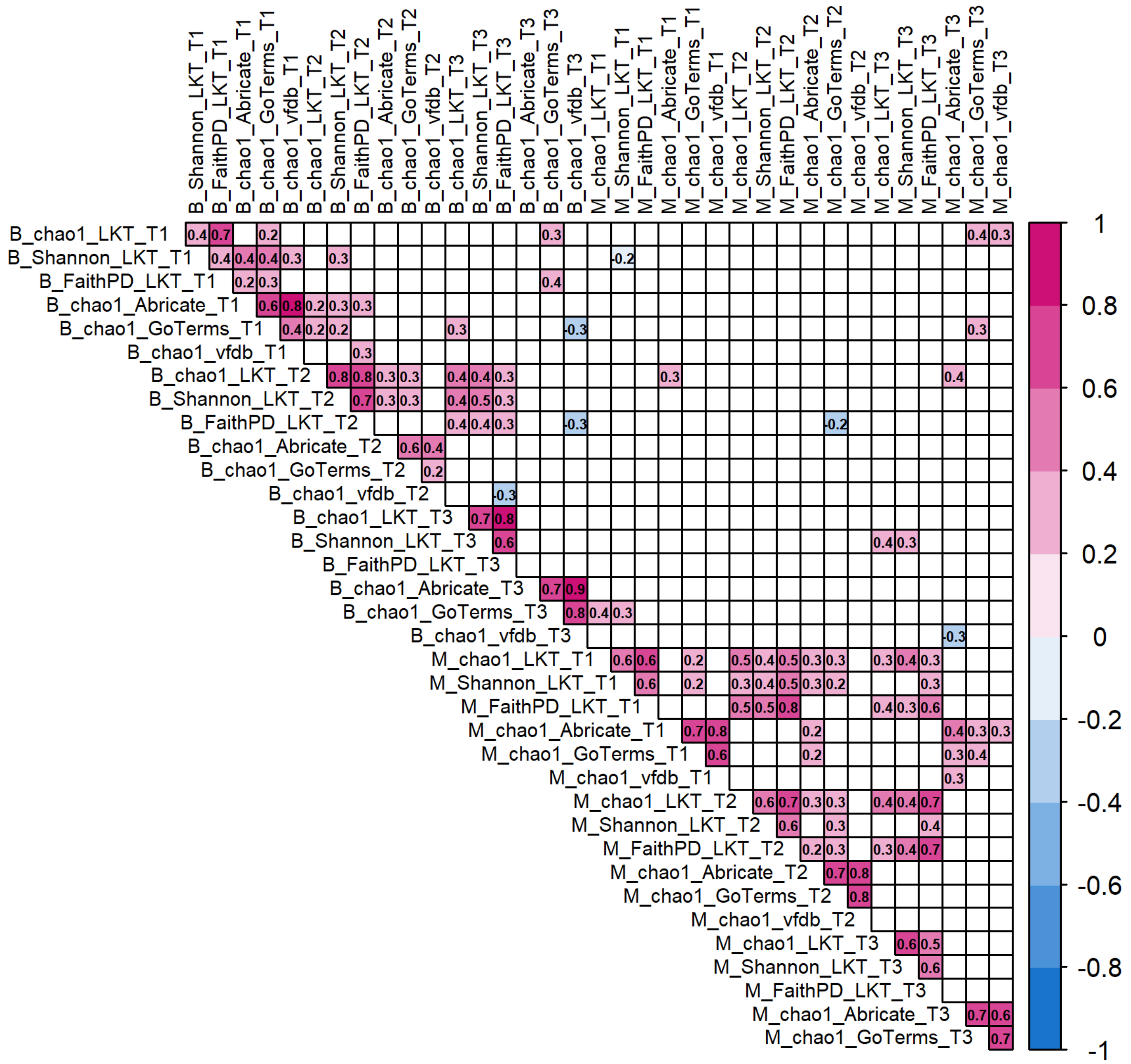

Note. Blank cells represent nonsignificant associations ($p > .05$). Abbreviations: Abricate – antibiotic resistance, B - Baby, LKT - Last Known Taxa, M - Mother, PD – Phylogenetic Diversity, T - Time, VFDB - Virulence Factor Database.

Supplementary Figure S8

Spearman's rank correlations between maternal and infant gut microbiomes at each time point.

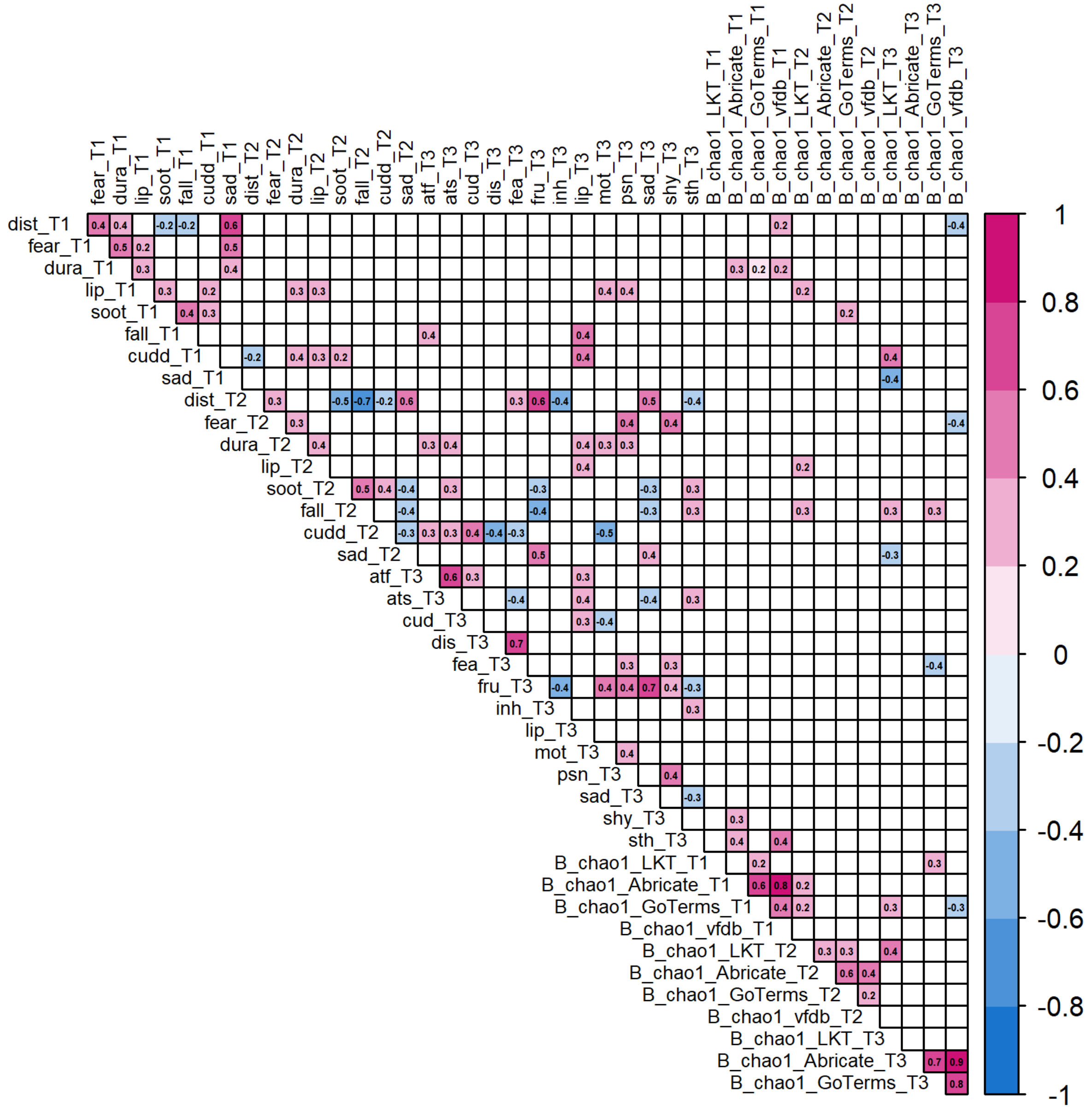

Note. Blank cells represent nonsignificant associations (p > .05). Abbreviations: Abricate – antibiotic resistance, B - Baby, LKT - Last Known Taxa, M - Mother, T - Time, VFDB - Virulence Factor Database, dist - distress to limitations, dura- Duration of Orienting, lip-Low Pleasure, soot-Soothability, fall-Falling Reactivity/Rate of Recovery from Distress, cudd-Cuddliness, sad-Sadness, atf - Attentional Focusing, ats - Attentional Shifting, cud -Cuddliness, dis-Discomfort, Fea-fear, fru -Frustration, inh-Inhibitory Control, mot-Motor Activation, psn - Perceptual Sensitivity, shy -Shyness, sth - soothability.

Supplementary Figure S9

Spearman's rank correlations between infant health assessed at age 7 months and maternal and infant gut microbiomes at each time point.

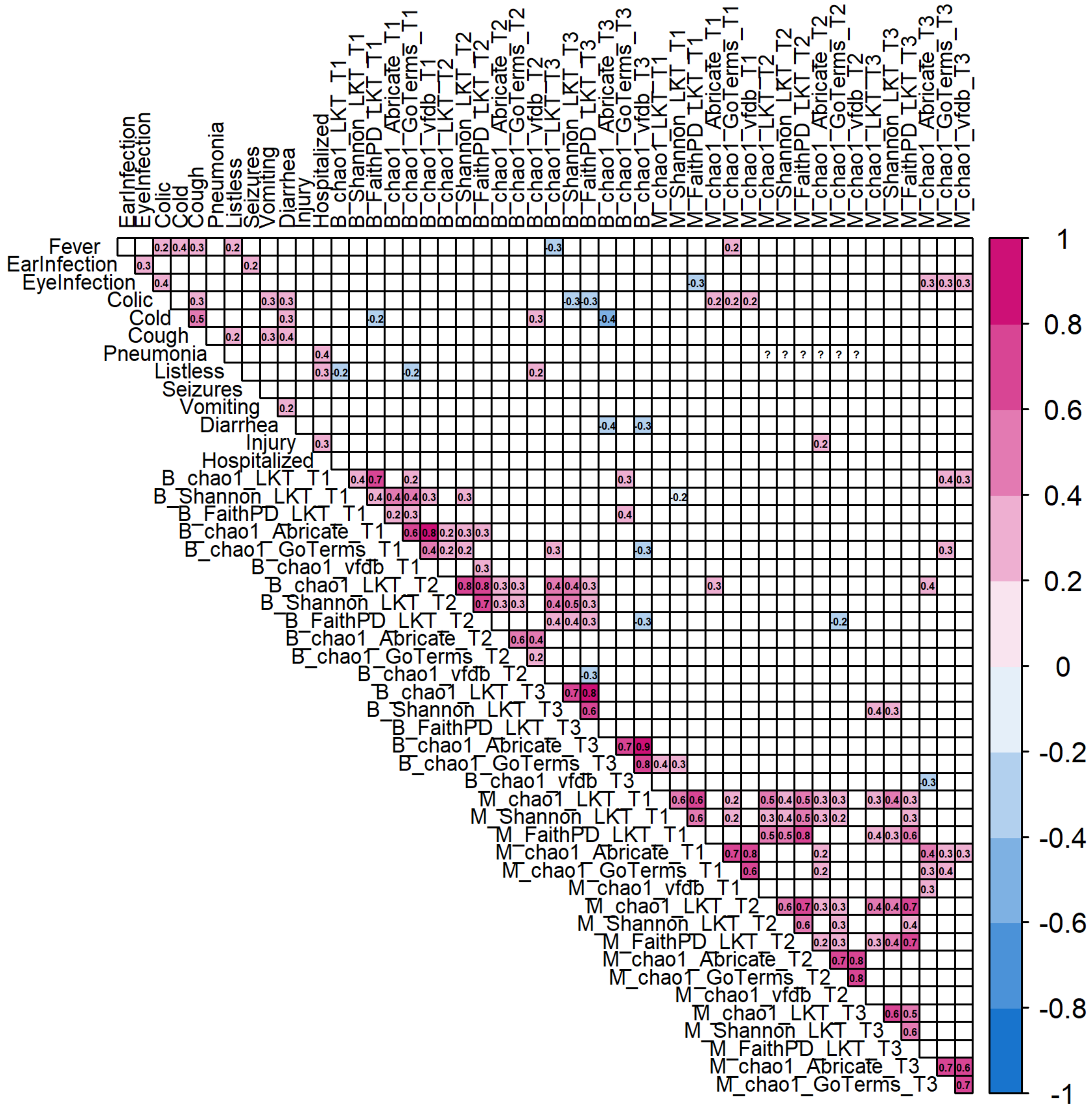

Note. Blank cells represent nonsignificant associations ($p > .05$) and ? indicates a lack of variability (none of the infants had pneumonia). Abbreviations: Abricate – antibiotic resistance, B - Baby, LKT - Last Known Taxa, M - Mother, T - Time, VFDB - Virulence Factor Database.

Supplementary Figure S10

Spearman's rank correlations between infant health assessed at age 7 months, infant temperament, and maternal depressive symptoms.

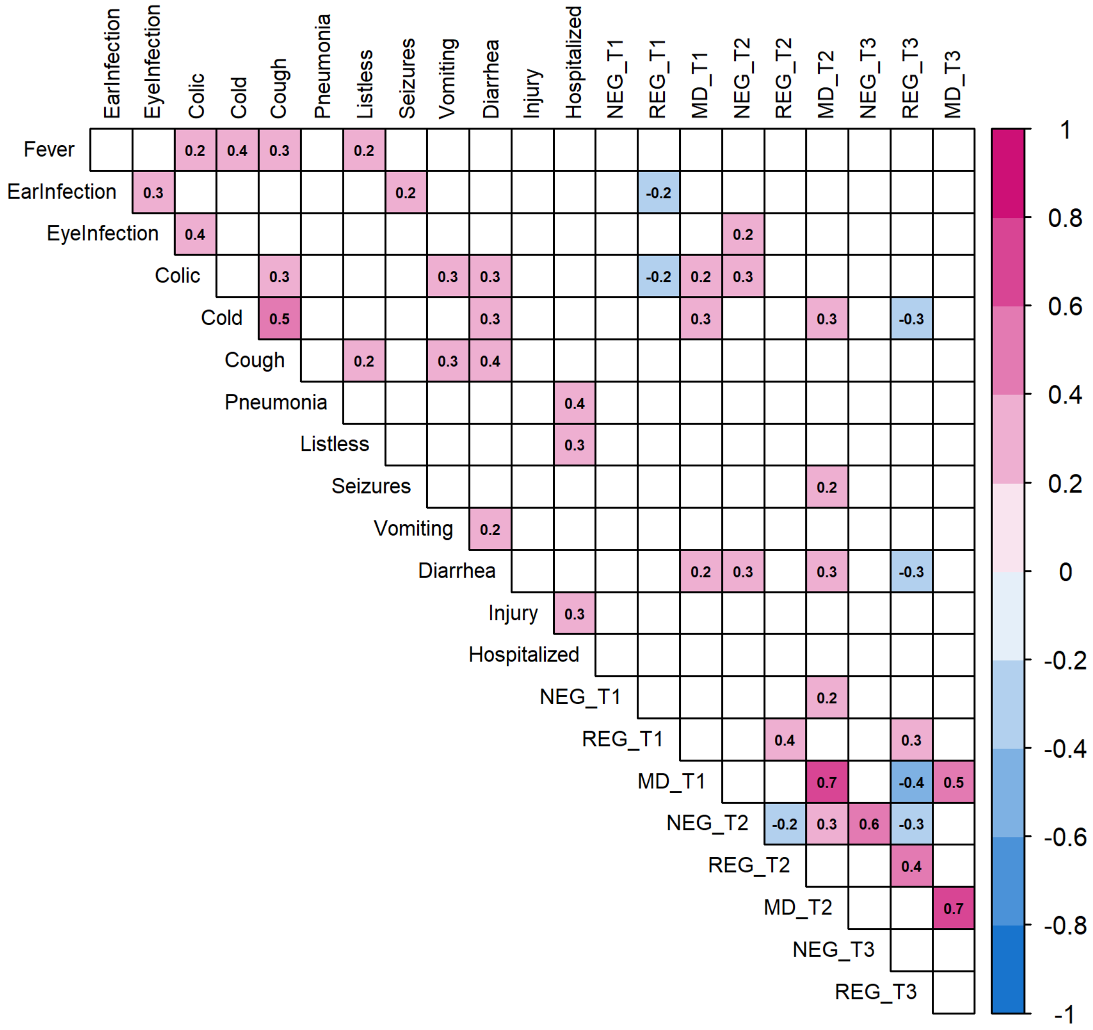

Note. Blank cells represent nonsignificant associations ($p >$ .05). Abbreviations: T - Time, NEG – negative affectivity, REG – regulation, MD – maternal depressive symptoms.

Supplementary Figure S11

Mother and infant Shannon taxa diversity over the first year of life.

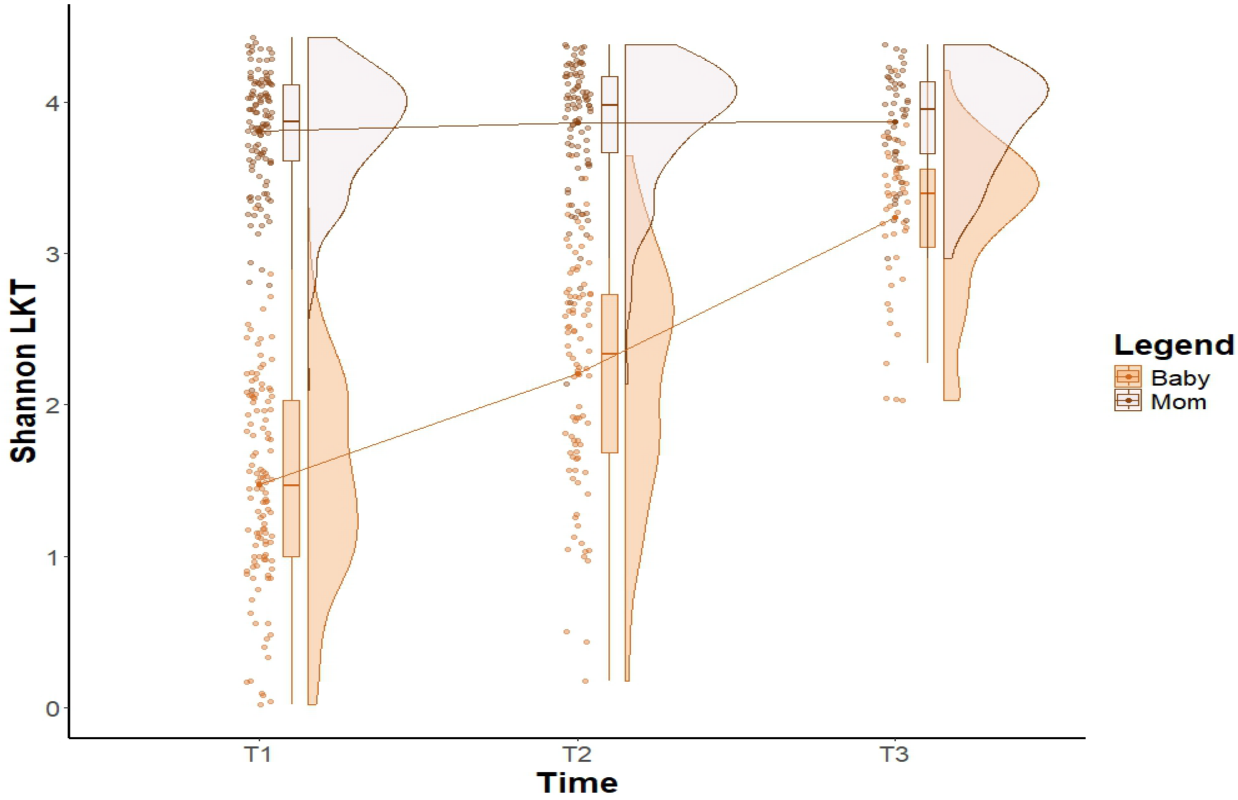

Note. Time 1 is approximately 1 month, T2 is approximately 7 months, and T3 is approximately 14 months. Abbreviations: T - Time.

Supplementary Figure S12

Mother and infant Faith's Phylogenetic Diversity over the first year of life.

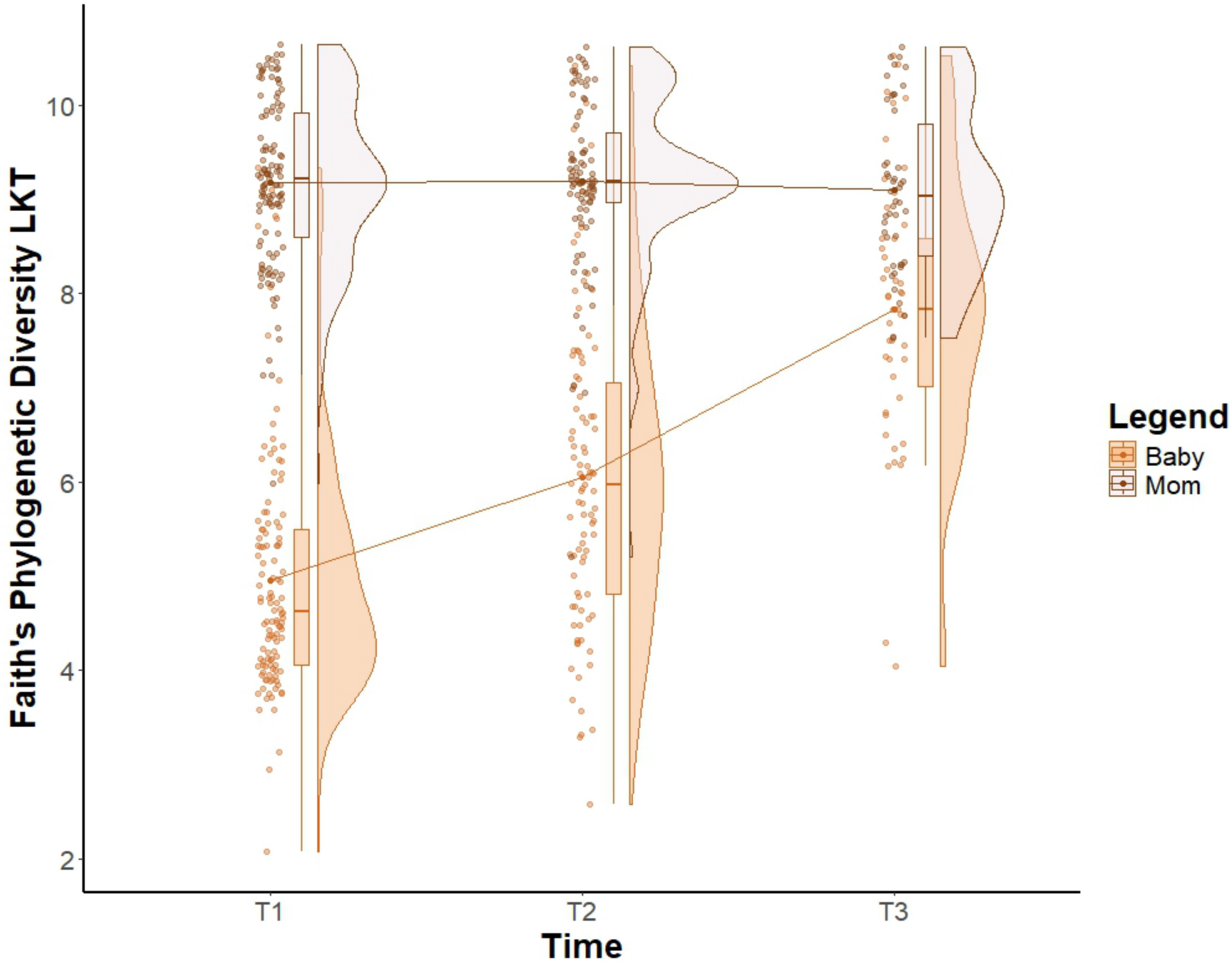

Note. Time 1 is approximately 1 month, T2 is approximately 7 months, and T3 is approximately 14 months. Abbreviations: T - Time.

Supplementary Figure S13

Mother and infant antibiotic resistance richness (Chao1 Abricate) over the first year of life.

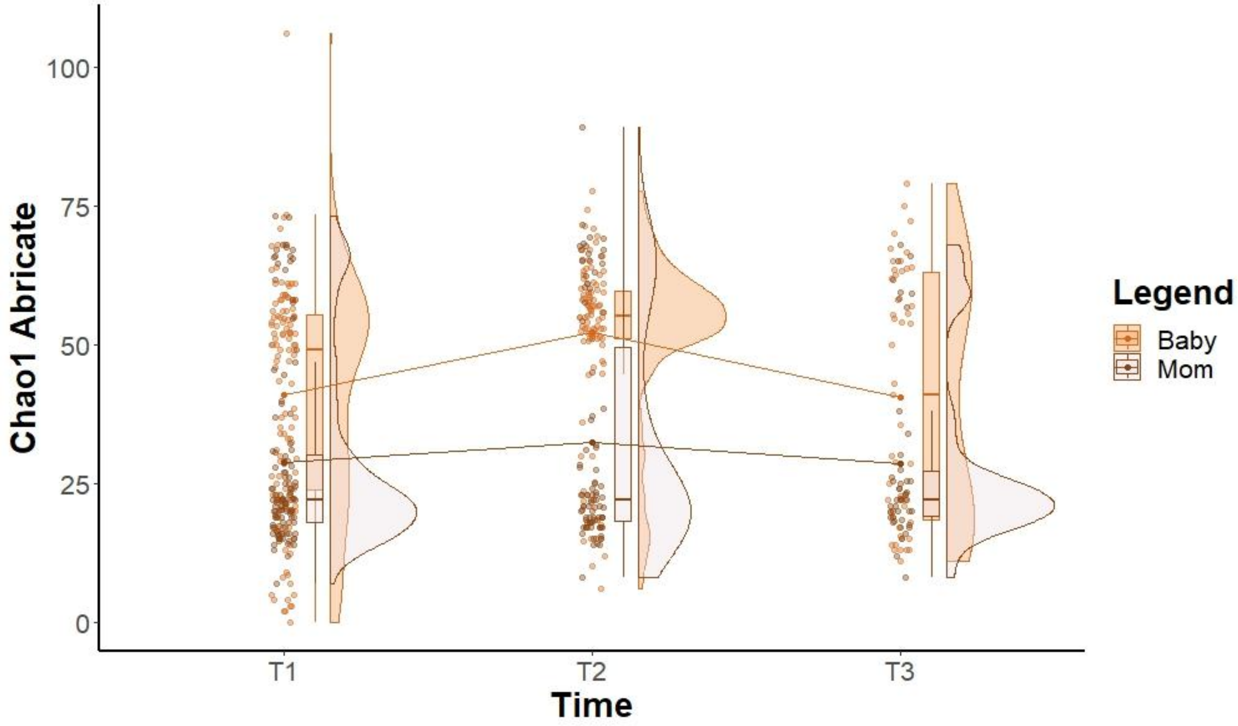

Note. Time 1 is approximately 1 month, T2 is approximately 7 months, and T3 is approximately 14 months. Abbreviations: T - Time.

Supplementary Figure S14

Mother and infant virulence factor richness (Chao1 VFDB) over the first year of life.

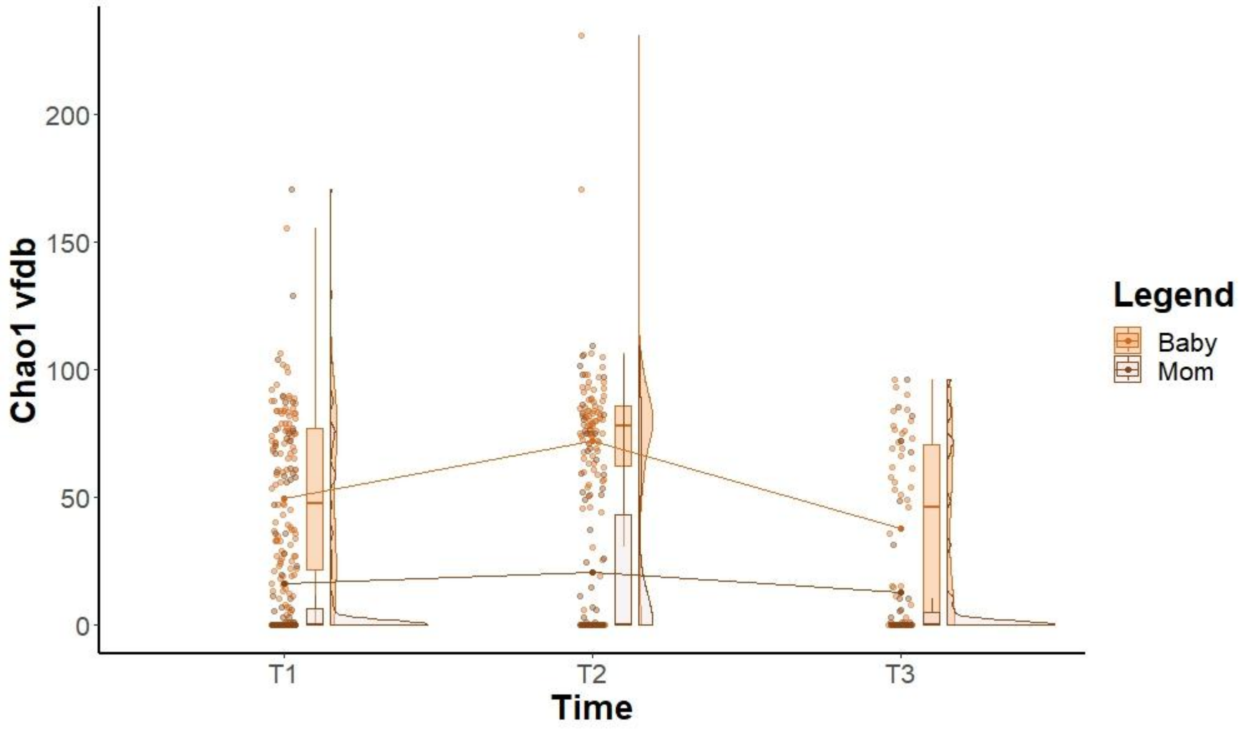

Note. Time 1 is approximately 1 month, T2 is approximately 7 months, and T3 is approximately 14 months. Abbreviations: T - Time, VFDB -virulence factor database.

Supplementary Figure S15

Mother and infant Gene Ontology richness (Chao1 GO) over the first year of life.

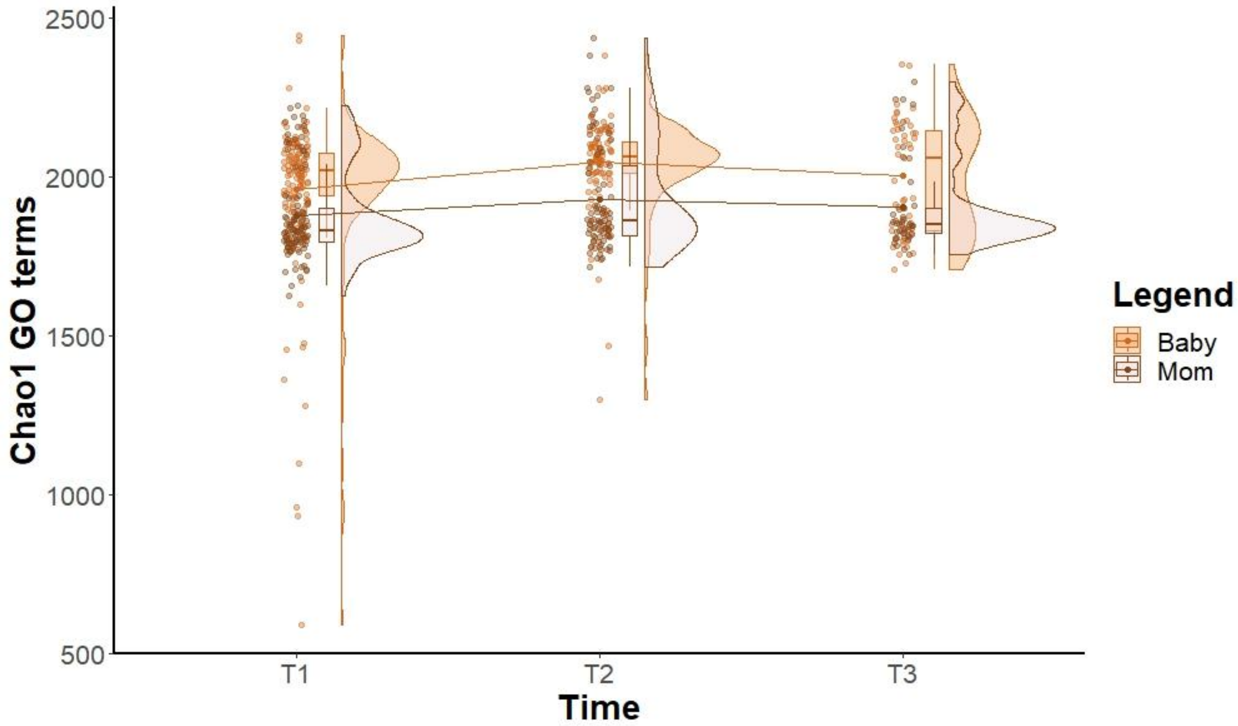

Note. Time 1 is approximately 1 month, T2 is approximately 7 months, and T3 is approximately 14 months. Abbreviations: T - Time.

Supplementary Tables

Supplementary Table S1

Covariate loadings from the principal component analysis.

Variable	PC1	PC2	PC3	PC4
M_Age_Birth	0.401	0.375	-0.491	0.176
C_Sex_Female	-0.178	0.016	0.244	0.016
Education	0.688	-0.099	-0.224	0.084
C_Race_NonWhite	-0.219	0.113	0.310	0.361
C-section	-0.165	0.428	-0.436	0.326
Gestational Age	0.278	-0.123	0.366	0.624
Birthweight	0.191	-0.019	-0.040	0.698
M_BMI_T1	-0.622	0.366	0.066	0.030
Antibiotics_duringlabor	-0.435	-0.117	0.145	0.170
M_antibiotics_afterbirth	-0.267	-0.273	0.552	-0.074
C_antibiotics_afterbirth	0.094	0.037	-0.210	-0.455
Breastfed_T2	0.692	-0.399	0.181	-0.132
Formula_T2	-0.638	0.141	-0.277	0.073
M_Diet_Fruits	0.201	0.708	0.372	-0.093
M_Diet_Vegetables	0.479	0.614	0.310	0.027
M_Diet_JunkFood	0.147	0.566	0.287	-0.277

Note. Abbreviations: PC-principal component analysis, M-mother, C-child, T-time, BMI-body mass index

Supplementary Table S2
Model estimates for associations between mother and infant chao1 LKT and psychological outcomes (maternal depression and negative emotionality).

See “Supplementary Table S2.xlsx”

Note. Parameters descriptions are intepreted by looking at the order of characters (e.g., B_M_GUT_GUT_T1_T2 is the path from infant gut microbiome at T1 to maternal gut microbiome at T2, whereas M_B_GUT_NEG_T1_T2 is the path from maternal gut microbiome at T1 to infant negative emotionality at T2). Abbreviations: M –mother, B- Baby, GUT -gut microbiome diversity assessment, T – Time, DEP- maternal depressive symptoms, NEG – negative emotionality, PC – principal component, CI – Credible Interval.

Supplementary Table S3
Model estimates for associations between mother and infant chao1 LKT and psychological outcomes (maternal depression and regulation).

See “Supplementary Table S3.xlsx”

Note. Parameters descriptions are intepreted by looking at the order of characters (e.g., B_M_GUT_GUT_T1_T2 is the path from infant gut microbiome at T1 to maternal gut microbiome at T2, whereas M_B_GUT_NEG_T1_T2 is the path from maternal gut microbiome at T1 to infant negative emotionality at T2). Abbreviations: M –mother, B- Baby, GUT -gut microbiome diversity assessment, T – Time, DEP- maternal depressive symptoms, REG – regulation, PC – principal component, CI – Credible Interval.

Supplementary Table S4
Model estimates for associations between mother and infant chao1 antibiotic resistance genes (abricate) and psychological outcomes (maternal depression and negative emotionality).

See “Supplementary Table S4.xlsx”

Note. Abbreviations: CI – Credible Interval, dep – depression, neg – negative emotionality, Sig – significance, T – Time.

Supplementary Table S5
Model estimates for associations between mother and infant chao1 antibiotic resistance genes (abricate) and psychological outcomes (maternal depression and regulation).

See "Supplementary Table S5.xlsx"

Note. Parameters descriptions are intepreted by looking at the order of characters (e.g., B_M_GUT_GUT_T1_T2 is the path from infant gut microbiome at T1 to maternal gut microbiome at T2, whereas M_B_GUT_NEG_T1_T2 is the path from maternal gut microbiome at T1 to infant negative emotionality at T2). Abbreviations: M – Mother, B- Baby, GUT -gut microbiome diversity assessment, T – Time, DEP- maternal depressive symptoms, REG – regulation, PC – principal component, CI – Credible Interval.

Supplementary Table S6
Model estimates for associations between mother and infant chao1 Gene Ontology (GO) Terms and psychological outcomes (maternal depression and negative emotionality).

See "Supplementary Table S6.xlsx"

Note. Parameters descriptions are intepreted by looking at the order of characters (e.g., B_M_GUT_GUT_T1_T2 is the path from infant gut microbiome at T1 to maternal gut microbiome at T2, whereas M_B_GUT_NEG_T1_T2 is the path from maternal gut microbiome at T1 to infant negative emotionality at T2). Abbreviations: M – Mother, B- Baby, GUT -gut microbiome diversity assessment, T – Time, DEP- maternal depressive symptoms, NEG – negative emotionality, PC – principal component, CI – Credible Interval.

Supplementary Table S7
Model estimates for associations between mother and infant chao1 Gene Ontology (GO) Terms and psychological outcomes (maternal depression and regulation).

See "Supplementary Table S7.xlsx"

Note. Parameters descriptions are intepreted by looking at the order of characters (e.g., B_M_GUT_GUT_T1_T2 is the path from infant gut microbiome at T1 to maternal gut microbiome at T2, whereas M_B_GUT_NEG_T1_T2 is the path from maternal gut microbiome at T1 to infant negative emotionality at T2). Abbreviations: M – Mother, B- Baby, GUT -gut microbiome diversity assessment, T – Time, DEP- maternal depressive symptoms, REG – regulation, PC – principal component, CI – Credible Interval.

Supplementary Table S8
Model estimates for associations between mother and infant chao1 virulence factor (vfdb) and psychological outcomes (maternal depression and negative emotionality).

See "Supplementary Table S8.xlsx"

Note. Parameters descriptions are intepreted by looking at the order of characters (e.g., B_M_GUT_GUT_T1_T2 is the path from infant gut microbiome at T1 to maternal gut microbiome at T2, whereas M_B_GUT_NEG_T1_T2 is the path from maternal gut microbiome at T1 to infant negative emotionality at T2). Abbreviations: M – Mother, B- Baby, GUT -gut microbiome diversity assessment, T – Time, DEP- maternal depressive symptoms, NEG – negative emotionality, PC – principal component, CI – Credible Interval.

Supplementary Table S9
Model estimates for associations between mother and infant chao1 virulence factor (vfdb) and psychological outcomes (maternal depression and regulation).

See "Supplementary Table S9.xlsx"

Note. Parameters descriptions are intepreted by looking at the order of characters (e.g., B_M_GUT_GUT_T1_T2 is the path from infant gut microbiome at T1 to maternal gut microbiome at T2, whereas M_B_GUT_NEG_T1_T2 is the path from maternal gut microbiome at T1 to infant negative emotionality at T2). Abbreviations: M – Mother, B- Baby, GUT -gut microbiome diversity assessment, T – Time, DEP- maternal depressive symptoms, REG – regulation, PC – principal component, CI – Credible Interval.

Supplementary Table S10
Infant gut microbiome taxa that were significant predictors of psychological variables.

See "Supplementary Table S10.xlsx"

Note: Abbreviations: s- identified at the species level, g- identified at the genus level, LKT - Last known taxa, MD - maternal depression, NEG - negative affectivity, REG – regulation, Log2fc – log 2 fold change.

Supplementary Table S11
Infant gut microbiome antibiotic resistance genes that were significant predictors of psychological variables.

See "Supplementary Table S11.xlsx"

Note. Abbreviations: MD - maternal depression, NEG - negative affectivity, REG – regulation.

Supplementary Table S12

Infant gut microbiome virulence factors that were significant predictors of psychological variables.

See “Supplementary Table S12.xlsx”

Note. Abbreviations: MD - maternal depression, NEG - negative affectivity, REG - regulation

Supplementary Table S13

Number of data points contributed across the 3 timepoints.

	Contributed 1 datapoint (*n*)	Contributed 2 datapoints (*n*)	Contributed 3 datapoints (*n*)
Individuals			
Infant microbiome	27	51	43
Infant temperament	30	53	37
Maternal microbiome	28	51	42
Maternal depressive symptoms	31	52	37
Dyads			
Microbiome	29	54	38
Behavior	31	52	37

Supplementary Table S14

Sample size for each of the data types.

	T1 (n)	T2 (n)	T2 (n)
Infant microbiome	120	91	47
Infant temperament	113	91	43
Maternal microbiome	119	91	46
Maternal depressive symptoms	112	91	43

Note. Abbreviations: T - Time

Supplementary Table S15
Relative abundances for all taxa identified in the sample.

See “Supplementary Table S15.xlsx”

Note. Abbreviations: LKT is Last Known Taxa, T - Time

Supplemental References

1 Kelsey, C. M. et al. Gut Microbiota Composition Is Associated with Newborn Functional Brain Connectivity and Behavioral Temperament. *Brain, Behavior, and Immunity* **91**, 472-486 (2021).

2 Mcculloch, J. *Just a Microbiology System (Jams)*, <https://github.com/johnmcculloch/JAMS_BW> (2023).

3 Davar, D. et al. Fecal Microbiota Transplant Overcomes Resistance to Anti–Pd-1 Therapy in Melanoma Patients. *Science* **371**, 595-602 (2021).

4 Dreisbach, C. et al. Composition of the Maternal Gastrointestinal Microbiome as a Predictor of Neonatal Birth Weight. *Pediatric Research* (2023).

5 Rosshart, S. P. et al. Laboratory Mice Born to Wild Mice Have Natural Microbiota and Model Human Immune Responses. *Science (New York, NY)* **365**, eaaw4361 (2019).

6 Bolger, A. M., Lohse, M. & Usadel, B. Trimmomatic: A Flexible Trimmer for Illumina Sequence Data. *Bioinformatics (Oxford, England)* **30**, 2114-2120 (2014).

7 Langmead, B. & Salzberg, S. L. Fast Gapped-Read Alignment with Bowtie 2. *Nat Methods* **9**, 357-359 (2012).

8 Li, D., Liu, C. M., Luo, R., Sadakane, K. & Lam, T. W. Megahit: An Ultra-Fast Single-Node Solution for Large and Complex Metagenomics Assembly Via Succinct De Bruijn Graph. *Bioinformatics (Oxford, England)* **31**, 1674-1676 (2015).

9 Wood, D. E. & Salzberg, S. L. Kraken: Ultrafast Metagenomic Sequence Classification Using Exact Alignments. *Genome biology* **15**, R46 (2014).

10 Seemann, T. Prokka: Rapid Prokaryotic Genome Annotation. *Bioinformatics (Oxford, England)* **30**, 2068-2069 (2014).

11 Ye, S. H., Siddle, K. J., Park, D. J. & Sabeti, P. C. Benchmarking Metagenomics Tools for Taxonomic Classification. *Cell* **178**, 779-794 (2019).

12 McIntyre, A. B. R. et al. Comprehensive Benchmarking and Ensemble Approaches for Metagenomic Classifiers. *Genome biology* **18**, 182 (2017).

13 *Interproscan*, <https://github.com/ebi-pf-team/interproscan> (2023).

14 Chen, L., Zheng, D., Liu, B., Yang, J. & Jin, Q. Vfdb 2016: Hierarchical and Refined Dataset for Big Data Analysis--10 Years On. *Nucleic Acids Res* **44**, D694-697 (2016).

15 Gupta, S. K. et al. Arg-Annot, a New Bioinformatic Tool to Discover Antibiotic Resistance Genes in Bacterial Genomes. *Antimicrob Agents Chemother* **58**, 212-220 (2014).

16 Feldgarden, M. et al. Validating the Amrfinder Tool and Resistance Gene Database by Using Antimicrobial Resistance Genotype-Phenotype Correlations in a Collection of Isolates. *Antimicrobial Agents and Chemotherapy* **63**, 10.1128/aac.00483-00419 (2019).

17 Jia, B. et al. Card 2017: Expansion and Model-Centric Curation of the Comprehensive Antibiotic Resistance Database. *Nucleic Acids Research* **45**, D566-D573 (2016).

18 Zankari, E. et al. Identification of Acquired Antimicrobial Resistance Genes. *J Antimicrob Chemother* **67**, 2640-2644 (2012).

19 Seemann, T. *Abricate*, <https://github.com/tseemann/abricate> (2020).

20 Putnam, S. P., Gartstein, M. A. & Rothbart, M. K. Measurement of Fine-Grained Aspects of Toddler Temperament: The Early Childhood Behavior Questionnaire. *Infant behavior and development* **29**, 386-401 (2006).

21 *Nhanes Food Frequency Questionnaire (Ffq)*, <https://epi.grants.cancer.gov/diet/usualintakes/ffq.html> (
22 Stekhoven, D. J. & Bühlmann, P. Missforest—Non-Parametric Missing Value Imputation for Mixed-Type Data. *Bioinformatics (Oxford, England)* **28**, 112-118 (2011).
